# A Single-Antenna RFID Machine Learning Approach for Direction and Orientation Tracking in Industrial Logistics

**DOI:** 10.3390/s26103144

**Published:** 2026-05-15

**Authors:** João M. Faria, Luis Vilas Boas, Joaquin Dillen, N. Simões, José Figueiredo, Luis Cardoso, João Borges, António H. J. Moreira

**Affiliations:** 1 2Ai—School of Technology, IPCA, 4750-810 Barcelos, Portugal; lvilasboas@ipca.pt (L.V.B.); jdillen@ipca.pt (J.D.); nsimoes@ipca.pt (N.S.); jpbsilva@ipca.pt (J.B.); 2 LASI—Associate Laboratory of Intelligent Systems, 4800-058 Guimarães, Portugal; 3 Sistrade—Software Consulting, 4250-380 Porto, Portugal; 4 Miranda & Irmão, 3750-871 Águeda, Portugal

**Keywords:** Radio Frequency Identification (RFID), machine learning (ML), deep learning (DL), tracking, domain adaptation, Internet of Things (IoT), logistics

## Abstract

Radio Frequency Identification (RFID) is an emerging technology in Industry 4.0 for low-cost logistics, yet direction and orientation estimation typically requires multiple antennas, and robustness under industrial multipath fading, operator variability, and signal fragmentation has not been evaluated. To address this gap, this study proposes a single-antenna RFID system that evaluated thirteen architectures spanning unsupervised methods (clustering algorithms) and supervised methods (classical machine learning, deep learning, and hybrid architectures) on Received Signal Strength Indicator (RSSI) and phase time-series reconstructed through a pipeline of Savitzky–Golay smoothing, phase unwrapping, and cubic spline resampling to N = 50–300 samples, preserving signal morphology across variable-length RFID passes. The system further incorporates a physics-informed augmentation strategy that encodes multipath fading, distance variation, and fragmentation into synthetic training samples for cross-domain generalization without hardware modification. In controlled laboratory experiments, both direction and orientation tasks achieved >99.5% accuracy, while direction tracking was additionally validated on an industrial shop floor under varying distances, Non-Line-of-Sight (NLoS) occlusions, and signal fragmentation. Zero-shot transfer caused accuracy to degrade to near-chance levels for several configurations, confirming a pronounced domain gap. Domain adaptation with XGBoost recovered direction accuracy to >97% under severe fragmentation under NLoS conditions, with an inference latency of ≈150 μs. Under domain-adapted shop floor conditions, direction accuracy exceeded the 75–92% reported in prior single-antenna laboratory studies, suggesting that physics-informed domain adaptation is a promising approach for single-antenna RFID tracking in Industrial Internet of Things (IIoT) logistics environments.

## 1. Introduction

Industry 4.0 is a subset of the Fourth Industrial Revolution, in which technologies such as robotics, automation, artificial intelligence, Internet of Things (IoT), and big data drive the industrial sector [1,2]. One of the emerging technologies in the industry is Radio Frequency Identification (RFID) [3]. Unlike traditional vision systems, RFID offers low-cost, non-line-of-sight (NLoS) communication and automated data collection [4,5], making it well-suited for supply chain management [6,7] and real-time production tracking [8,9].

Despite this widespread adoption, significant challenges remain in estimating the position and orientation of passive RFID tags [10,11,12]. Although multiple-antenna configurations achieve high accuracy, this approach significantly increases hardware costs, system complexity, and installation constraints. Furthermore, even multi-antenna configurations frequently struggle to meet the reliability standards required for automated palletization, robotic handling, and collision-free logistics. In the specific domain of direction estimation, a preliminary single-antenna approach [13] demonstrated theoretical feasibility but lacked algorithmic robustness and validation in real shop floor environments.

In summary, current single-antenna RFID solutions for direction and orientation tracking lack three key properties simultaneously: (i) industrial-grade accuracy for both movement direction and static box orientation; (ii) physics-informed cross-domain adaptation strategies that explicitly target the gap between clean laboratory signals and fragmented shop floor data; and (iii) systematic comparisons of unsupervised (clustering) and supervised learning (classical machine learning, deep learning, and hybrid architectures) under industrial conditions.

To address these limitations, this study proposes a single-antenna RFID system for estimating the movement direction and orientation of passive tags attached to a logistics box. Specifically, this study addresses three research objectives: (i) whether complete-pass morphological representations of RFID signals, reconstructed via a unified preprocessing pipeline, can overcome the accuracy limitations of prior short-segment approaches for the simultaneous estimation of direction and orientation; (ii) whether a data augmentation strategy grounded in electromagnetic propagation models can enable effective cross-domain transfer from controlled laboratory conditions to unstructured industrial shop floor environments without additional hardware; and (iii) which learning paradigm, namely unsupervised clustering, classical supervised learning, deep learning, or hybrid architectures, offers the best trade-off between accuracy, robustness to signal fragmentation, and inference latency for edge deployment in IIoT logistics.

The main contributions of this paper are:

- A unified single-antenna preprocessing pipeline that combines Received Signal Strength Indicator (RSSI) smoothing using a Savitzky–Golay filter, phase unwrapping, and cubic spline interpolation to produce fixed-length signal representations while preserving the morphology of variable-length RFID tags.
- A physics-informed data augmentation (DA) strategy that synthetically replicates shop floor noise conditions during training, enabling robust cross-domain generalization without additional hardware.
- A systematic evaluation of thirteen machine learning architectures, covering unsupervised (clustering) and supervised (classical ML, deep learning, and hybrid architectures), under identical conditions in controlled laboratory experiments for both direction and orientation, and targeted shop floor tests for direction tracking.

From an application perspective, the proposed system enables companies to optimize internal logistics through continuous, automated direction monitoring at RFID checkpoints, while simultaneously improving orientation control during transport and distribution. The integration with Manufacturing Execution Systems (MES) and Enterprise Resource Planning (ERP) platforms ensures that items pass through production stages with the correct orientation, updating inventory and manufacturing tracking. This contributes directly to the reliability of automated palletization, robotic handling, and collision-free material flow. The shift of analytical intelligence from multi-antenna hardware to AI models running on commodity RFID readers reduces dependence on costly computer vision alternatives, which remain sensitive to lighting and occlusion, positioning the proposed system as a deployable software-defined upgrade for existing single-antenna passive UHF RFID infrastructure in Industry 4.0 and Industrial Internet of Things (IIoT) logistics environments.

The remainder of this paper is organized as follows: Section 2 reviews the state of the art and identifies the specific limitations motivating this work. Section 3 describes the experimental setup, preprocessing pipeline, data augmentation strategy, and modeling approaches. Section 4 presents the experimental results, followed by discussion in Section 5. Section 6 presents the conclusions and future work.

## 2. Related Work

RFID technology originated during World War II [14] and has since attracted continuous research, with applications spanning a wide range of fields [15]. One of the main research areas is the estimation of position, orientation, and motion of RFID tags. This section reviews key approaches in multi-antenna RFID localization and tracking (Section 2.1), single-antenna direction estimation (Section 2.2), and the integration of machine learning and deep learning with RFID signal processing (Section 2.3). Finally, Section 2.4 synthesizes the limitations of existing methods and positions the present work within this landscape.

### 2.1. Multi-Antenna RFID Systems for Position and Orientation Estimation

Several works have demonstrated high-accuracy RFID localization and orientation tracking using multi-antenna configurations. Systems such as R-SHOT [10] and OmniTrack [11] employ two antennas with RSSI and phase-based models to estimate head orientation and object tracking, while other approaches combined MUSIC with phase-based models [12] and particle filter tracking over 14 switched antennas [16], achieving precise positioning in industrial supply chains. Trajectory reconstruction via Gaussian processes with 8-antenna arrays [17] and hybrid Time-of-Flight (ToF)/Angle-of-Arrival (AoA) radar fusion [18] demonstrated the scope of multimodal approaches. At the hardware frontier, dedicated full-array architectures for real-time AoA estimation [19] and tag-array AoA systems with explicit mutual coupling compensation [20] demonstrated high angular resolution, albeit requiring specialized Radio Frequency (RF) front-ends and extensive offline calibration procedures. Despite their high accuracy in controlled settings, the reliance on multiple antennas, auxiliary sensors, or dedicated hardware severely limits real-world scalability. This hardware dependency drives up deployment costs and introduces complex calibration overheads that are impractical for most industrial deployments.

### 2.2. Single-Antenna RFID Direction and Orientation Estimation

To overcome the hardware constraints and calibration overhead of multi-antenna systems, recent research has explored single-antenna configurations. For instance, Gil-Martinez et al. [21] proposed a cost-effective approach using a passive beam-scanning leaky-wave antenna for Ultra High Frequency (UHF) tag direction finding. While hardware-efficient, its capability to provide continuous, real-time tracking of moving tags in dynamic environments remains unclear. Addressing real-time motion tracking with a standard single commercial antenna, Faria et al. [13] demonstrated that classical classifiers could analyze short sequences of consecutive phase and RSSI samples from a laboratory conveyor to distinguish leftward from rightward motion (binary output), achieving accuracy of 75–90%, but without shop floor validation or data augmentation strategies. In a parallel effort focused on box orientation, Dillen et al. [22] utilized short fixed-length sequences (up to 9 samples per tag) of phase and RSSI data in a controlled laboratory environment to classify three box orientations, achieving up to 92% accuracy using the LightGBM ensemble, but restricted to a single model family and without cross-domain evaluation.

Taken together, these works established the feasibility of single-antenna direction and orientation estimation, but left three key questions open: (i) how robust such systems remain under severe multipath and operational variability on industrial shop floors, (ii) whether higher accuracy can be achieved when modeling complete RFID passes instead of short segments, and (iii) how clustering, classical ensembles, deep learning, and hybrid architectures compare under identical conditions.

### 2.3. Machine and Deep Learning for RFID Signal Processing

Artificial intelligence has fundamentally transformed various industrial sectors by streamlining complex processes and enhancing operational efficiency [23]. Despite the broad adoption of AI in industrial sensing, RFID-specific AI application remains limited, with most studies confined to controlled laboratory conditions and lacking systematic paradigm comparisons. Early applications of AI to RFID primarily targeted ambient-assisted living (AAL) and indoor localization. Oguntala et al. [24] used RSSI time series from two passive RFID antennas as input to an LSTM, achieving over 98% accuracy in human activity recognition (e.g., sitting, walking), a multi-class classification task in which temporal signal dynamics serve as the primary discriminative cue. For spatial localization, Peng et al. [25] combined RSSI and Phase Difference of Arrival (PDOA) fingerprints as inputs to a deep CNN to estimate static indoor tag positions, outperforming classical fingerprinting methods in multipath-rich environments. More recently, Huang et al. [26] proposed an LSTM-CNN hybrid for RFID-based IoT device identification, demonstrating the advantage of combining convolutional feature extraction with sequential modeling for RFID time-series classification tasks.

In the broader context of IoT signal classification, tree-based ensemble methods such as XGBoost have been widely adopted due to their resistance to overfitting and capacity to handle non-linear feature interactions [27]. For single-antenna direction and orientation estimation specifically, no systematic comparison between classical machine learning, deep learning, and hybrid architectures has been reported in the literature. Equally absent are physics-informed DA strategies that explicitly encode the multipath and fragmentation characteristics of industrial environments, as prior work relies exclusively on data collected under controlled settings. While data augmentation grounded in physical propagation models has been explored in adjacent RF sensing domains, no prior RFID work has grounded augmentation in the indoor path loss and backscatter propagation models to bridge the gap between clean laboratory signals and fragmented industrial acquisitions. Reported accuracy values for these tasks (75–92%) are insufficient for reliable deployment in logistics operations such as automated parcel sorting, robotic palletizing, and collision avoidance, where error rates directly translate into operational failures.

### 2.4. Research Gap and Positioning of the Present Work

The literature reviewed in this section highlights three limitations motivating the present study:

1. Hardware cost and complexity: Most high-accuracy RFID localization and orientation solutions rely on multiple antennas, including full-array AoA estimation systems, which increase hardware cost, installation effort, and calibration overhead. Single-antenna approaches remain comparatively underexplored, particularly for direction and orientation estimation.
2. Insufficient robustness for industrial operation: AI-based RFID orientation and motion inference typically reports 75–92% accuracy, which is inadequate for safety-critical logistics tasks (e.g., palletizing) that demand high reliability.
3. Lack of real-world validation: Most studies are evaluated exclusively in controlled laboratory environments. Industrial shop floor conditions, characterized by multipath interference, operator-induced variability, and signal fragmentation, remain largely absent from the validation literature. This raises fundamental questions about the actual deployability of existing solutions, since signal distributions in unstructured industrial environments differ substantially from those observed under controlled acquisition, and models trained exclusively on clean signals are unlikely to generalize without explicit domain adaptation.

To address these gaps, this work proposes a single-antenna RFID system with a unified preprocessing pipeline that reconstructs RFID passes from phase and RSSI time series into fixed-length, morphology-preserving signal representations, and a physics-informed data augmentation strategy that synthetically replicates shop floor noise conditions to improve model cross-domain robustness. Within this framework, the evaluation encompasses a systematic comparison of unsupervised learning (clustering) and supervised learning (classical ML, deep learning, and hybrid architectures) under identical preprocessing conditions. Validation spans both controlled conveyor experiments and real industrial shop floor trials, enabling a realistic assessment of robustness for Industry 4.0/IIoT logistics.

## 3. Materials and Methods

**Figure 1:**
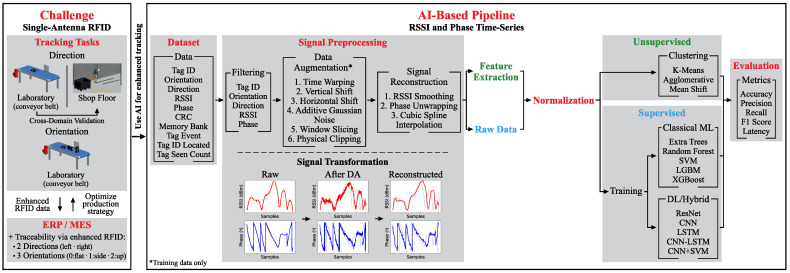
Overview of the proposed AI-based single-antenna RFID pipeline for product direction and orientation tracking in industrial logistics. The Challenge block illustrates the two estimation tasks (direction and orientation), and their integration with ERP/MES traceability. The AI-Based Pipeline encompasses dataset acquisition, physics-informed data augmentation, signal reconstruction, and systematic evaluation across unsupervised and supervised approaches.

This section describes the methodological pipeline developed to estimate movement direction and box orientation from a single-antenna RFID setup, integrating the unified preprocessing pipeline, the physics-informed data augmentation strategy, and the systematic model evaluation framework. Figure 1 illustrates the complete workflow, and the following subsections detail each component.

### 3.1. Experimental Setup and Data Acquisition

To evaluate the proposed methodology under varying operational conditions and complexity levels, the experiments encompassed two distinct scenarios, namely a controlled laboratory environment using an industrial conveyor belt, and a real-world validation scenario on a shop floor using a manual pallet truck. Both setups utilized a Zebra AN510 RFID antenna (Zebra Technologies Corp., Lincolnshire, IL, USA) (8.5 dBic gain, right-hand circular polarization) [28] connected to a Zebra FX9600 reader (Zebra Technologies Corp., Lincolnshire, IL, USA) [29] to capture RF signal parameters (Figure 1—Dataset).

#### 3.1.1. Laboratory Setup

**Figure 2:**
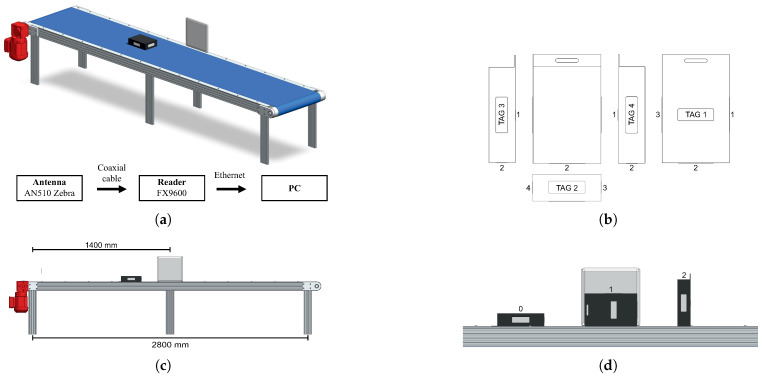
Laboratory Setup. (**a**) General view of the dataset collection system. (**b**) Front view of the dataset collection system. (**c**) Dimensions of the box with 4 labels centered on different sides. (**d**) Example of the three orientations (0, 1 and 2) studied on the conveyor belt.

A repeatable and configurable system was developed to simulate material transport between industrial sections and logistics distribution chains, where reliable box positioning is critical for automated downstream handling and production traceability. The setup consisted of an industrial conveyor belt, the RFID hardware, and a product box measuring 200×135×55 mm, which enclosed a component made of an aluminum–iron alloy to introduce realistic metallic interference, as shown in Figure 2a. The box contained four passive RFID tags (75×25 mm) intentionally placed on different faces (Figure 2b) without enforcing precise geometric alignment, to assess robustness under non-ideal deployment conditions. The compact box dimensions placed adjacent-face tags in close physical proximity, deliberately introducing near-field electromagnetic interference, including mutual coupling and antenna detuning, effectively acting as a worst-case stress test for the evaluated models.

Data acquisition targeted two specific tasks. The first task required detecting movement direction, while keeping a single box orientation fixed (Figure 2c), and the second required estimating orientation across three distinct configurations (Figure 2d). Each combination of direction, orientation, and speed was repeated 100 times at three controlled speeds (0.20 m/s, 0.40 m/s, and 0.80 m/s) along a 2800 mm path (Figure 2c), yielding 600 passes per tag for direction estimation (2 directions × 3 speeds × 100 repetitions) and 1800 passes per tag for orientation estimation (3 orientations × 3 speeds × 100 repetitions × 2 directions). The acquisition system stored all data in CSV format, organized by task parameters for subsequent processing. To ensure data integrity, only readings from RFID tags registered in the company’s ERP/MES system were retained, thereby limiting processing to the target product during active transport.

#### 3.1.2. Industrial Shop Floor Setup

**Figure 3:**
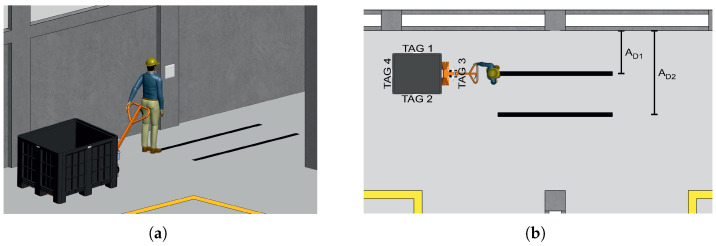
Industrial Shop Floor Setup. (**a**) Overview of the test system on the shop floor. (**b**) Top view, where AD1 and AD2 represent the distance from the box pallet to the antenna with the respective position of the tags.

To validate the system in a real-world environment, experiments were conducted on an industrial shop floor (Figure 3a), with the RFID antenna positioned at 1 m height. An operator pulled a pallet truck (1.2×1×0.83 m) carrying a plastic pallet box with tags in front of the antenna.

Unlike the controlled laboratory setup, this experiment introduced human-induced variability in movement speed and trajectory. The evaluation covered two antenna distances, 1 m (A_D1_) and 2 m (A_D2_), to assess performance under different signal propagation conditions (Figure 3b). Data collection yielded 290 and 220 passes per direction per tag at 1 m and 2 m, respectively, reflecting the operational constraints of the industrial environment. This dataset served two purposes: to evaluate the zero-shot transfer of laboratory-trained models to industrial conditions, and to provide domain-specific training data for shop floor adaptation.

This setup focused specifically on validating the direction detection capability of the best-performing models identified in the laboratory phase, serving as a stress test for the proposed system under uncontrolled industrial conditions.

### 3.2. Signal Preprocessing

Raw RFID data streams exhibited inherent irregularities, including varying sampling rates, transient noise, and communication gaps. The preprocessing pipeline addressed these irregularities by sequentially executing initial data filtering, physics-informed data augmentation (applied exclusively to training sets), temporal reconstruction for uniform sampling, and robust signal segmentation for shop floor validation.

#### 3.2.1. Filtering

The first stage of dataset processing involved filtering the raw data to remove redundant or non-informative features. The retained fields were the tag identification, box orientation label, movement direction label, RSSI, and phase (Figure 1—Preprocessing).

#### 3.2.2. Data Augmentation Strategy

To ensure robust generalization, a physics-informed data augmentation strategy addressed the physical differences between deployment environments through two complementary protocols. The first, a *Variance-based Protocol* for controlled laboratory baselines, injected synthetic noise to degrade pristine signals and prevent overfitting. The second, a *Probabilistic Geometric Protocol* for shop floor adaptation, relied exclusively on spatiotemporal transformations to preserve already degraded organic signals. Both protocols operated exclusively on raw, non-resampled training data to preserve temporal morphology. To prevent data leakage, testing was strictly performed on original, non-augmented samples.

##### Scenario-Based Signal Characterization

Rather than applying uniform perturbations, the augmentation strategy calibrated intensity to the intrinsic noise of each passage. Given that the physical manifestations of signal degradation differ between motion tracking and static pose detection, the scenario assignment strategy tailored the augmentation to each specific task:

- Direction Estimation (Environmental Profiling): Since the box maintained a constant pose, signal variability was primarily driven by external multipath fading and operator speed. Tag 2, empirically identified as the most stable tag facing the antenna trajectory, served as an environmental probe, with its raw RSSI standard deviation ($\sigma_{RSSI}$) dictating the noise scenario applied uniformly to all four tags. Applying the same scenario uniformly preserved temporal coherence and relative phase offsets between tags, both critical for directionality.
- Orientation Estimation (Global Pose Profiling): Box rotation inherently subjects different tags to varying degrees of line-of-sight (LoS) and severe NLoS occlusion due to the internal metallic component. Relying on a single reference tag would introduce spatial bias (e.g., classifying a passage as extremely noisy simply because the reference tag was on the shadowed face). To prevent this, the aggregate $\sigma_{RSSI}$ computed from all tag readings within the passage determined the noise scenario, providing an aggregate, pose-independent measure of the signal-to-noise ratio.

Based on the task-specific $\sigma_{RSSI}$ quartile distribution, each passage was assigned to one of four noise scenarios (A to D, Table 1).

##### Augmentation Pipeline

For each training sample, the augmentation pipeline generated three synthetic copies independently (a 4× expansion) by sequentially applying six transformations to the raw signals:

1. Time Warping: Non-uniform temporal distortion applied a symmetric Beta distribution (α) to simulate variability in the tag reading rate, with linear interpolation reconstructing the warped signal over the original sample grid.
2. Vertical Shift (Distance Variation): A random displacement $\Delta d∼U(-d_{max},+d_{max})$ cm was applied to simulate antenna-to-tag distance variability. The RSSI perturbation followed the indoor path loss model:
  (1) $$\Delta RSSI=-10\;n\log_{10}\;\left(\frac{d_{ref}+\Delta d}{d_{ref}}\right)$$
  where n=2.5 is the indoor path loss exponent and $d_{ref}=50$ cm is the nominal reference distance. The corresponding phase perturbation followed the RFID backscatter propagation model:
  (2) $$\Delta \phi =\frac{4\pi \;\Delta d}{\lambda }$$
  where λ=32.7 cm is the carrier wavelength at 915 MHz.
3. Horizontal Shift: Slope-based linear extrapolation padding at signal edges (start, end, or both) to simulate reading window misalignment without artificial discontinuities.
4. Additive Gaussian Noise: Zero-mean noise added to RSSI ($\sigma_{r}$) and phase ($\sigma_{\phi }$) to replicate RF measurement uncertainty.
5. Window Slicing: Random removal of 10% to 20% of edge samples (probability $p_{w}$) to simulate incomplete readings caused by tag occlusion or variable operator speed.
6. Physical Clipping: RSSI values constrained to the reader’s operational range [−85,−25] dBm.

The augmentation parameters operated at three levels: (i) physics-derived (n=2.5, λ=32.7 cm, and the [−85,−25] dBm clipping range) fixed by the propagation model and reader specifications; (ii) empirically calibrated scenario thresholds (Table 1), derived from the quartile distribution of $\sigma_{RSSI}$ across the collected dataset; and (iii) heuristically tuned parameters (α, $d_{max}$, $p_{w}$), selected to maximize training diversity while keeping synthetic signals within the physical operational envelope enforced by the clipping step.

As detailed in Table 1, the Gaussian noise injection strategy ($\sigma_{r}$ and $\sigma_{\phi }$) followed a task-specific design calibrated to the physical characteristics of each tracking task. For direction estimation, an inverse-proportional strategy deliberately assigned stronger noise perturbations to cleaner signals (Scenario A) relative to moderately noisy ones (Scenario B), on the premise that models trained predominantly on ideal conditions are unlikely to generalize to noisier industrial environments. Conversely, for orientation estimation, signal degradation is primarily driven by physical occlusion (NLoS) from box rotation rather than environmental variance, justifying a direct-proportional strategy. This task-specific domain randomization encouraged the models to learn invariant morphological signatures of motion.

##### Probabilistic Augmentation for Unstructured Constraints

The variance-based protocol (Table 1) relied on identifiable “clean” baselines, making it unsuitable for highly degraded industrial datasets where severe multipath distortion is ubiquitous. To support these unstructured environments, a secondary probabilistic protocol handled signal variability without relying on clean baselines (Table 2). In this approach, additive Gaussian noise was omitted ($\sigma_{r}=0$ and $\sigma_{\phi }=0$) to avoid masking the native, fragmented kinematic features. The augmentation focused exclusively on temporal and spatial variability to accurately model human-induced trajectory deviations and speed fluctuations. All training samples underwent probabilistic transformations across three severity levels (Very Mild, Mild, Moderate), exposing the models to realistic geometric shifts without degrading the underlying signal-to-noise ratio.

After the data augmentation, all signals, original and synthetic, passed through the full resampling pipeline described in Section 3.2.3, ensuring identical preprocessing for all training samples regardless of their origin.

#### 3.2.3. Signal Reconstruction

During the box’s passage in front of the antenna, signals from the four tags were acquired at varying rates due to differences in distance, angle, and potential obstructions, yielding a variable number of samples per tag. Since machine learning models require fixed-length input, each signal was standardized to *N* samples (ranging from 50 to 300, as illustrated in Figure 1—Preprocessing) through a time-domain reconstruction approach that preserves transient signal morphology and minimizes interpolation artifacts. This reconstruction proceeded in three steps:

1. RSSI Smoothing: The raw RSSI signal was smoothed using a Savitzky–Golay filter [30] (window length = 31, polynomial order = 3), preserving relative maxima, minima, and width that are critical for motion characterization, while reducing high-frequency noise.
2. Phase Unwrapping: The raw phase signal, originally wrapped in the [−π,π] interval, was unwrapped to remove the artificial 2π discontinuities introduced by the reader’s phase output, ensuring a continuous function as a prerequisite for accurate interpolation.
3. Cubic Spline Interpolation: Both the smoothed RSSI and the unwrapped phase were resampled to *N* via Cubic Spline interpolation with natural boundary conditions. The resulting piecewise third-order polynomial guaranteed continuity of the first and second derivatives, a property critical for feature extraction.

Finally, the resampled unwrapped phase $\phi_{unwrapped}$ was re-wrapped to the [−π,π] interval (and converted to degrees) to maintain consistency with standard RFID reader outputs, as described by:

(3) $$\phi_{wrapped}\left[n\right]=\mathrm{angle}\left(\exp(j\cdot \phi_{unwrapped}\left[n\right])\right)$$

where $\phi_{unwrapped}\left[n\right]$ represents the resampled unwrapped phase at sample *n*.

This process ensured that all input vectors for the machine learning models were temporally aligned and free from sampling-rate induced variations.

#### 3.2.4. Robust Signal Segmentation and Gap Analysis for Shop Floor

Unlike the controlled laboratory environment, the industrial shop floor introduced significant noise, including spurious readings, tag occlusion, and signal interruptions due to operator movement variability. To address this, a burst detection algorithm trimmed unstable leading and trailing edges by scanning the timestamp sequence for the longest continuous segment in which the inter-arrival time remained below 150 ms. This segment defined the stable communication window for subsequent signal quality characterization. Four interval levels classified the signal based on the maximum inter-arrival gap $G_{max}$ observed within the trimmed burst:

- Level 0 (Clean): Continuous signals with minimal interruptions ($G_{max}<500$ ms).
- Level 1 (Minor Gaps): Signals with short interruptions ($500\leq G_{max}<1000$ ms).
- Level 2 (Moderate Gaps): Signals with noticeable fragmentation ($1000\leq G_{max}<1500$ ms).
- Level 3 (Severe Gaps): Highly fragmented signals with large interruptions ($G_{max}\geq 1500$ ms).

This gap-based classification enabled fine-grained performance analysis, quantifying how model accuracy declined as signal continuity deteriorated under real-world conditions.

### 3.3. Feature Extraction and Selection for Clustering Analysis

Effective unsupervised clustering requires transforming the high-dimensional time-series data into a compact set of discriminative features. Unlike classical machine learning or deep learning models, which in this study processed raw signals directly, clustering algorithms require a feature vector that captures the signal’s morphological, statistical, and spectral characteristics.

#### 3.3.1. Feature Extraction

From the resampled RSSI and phase signals, each pass was represented by a comprehensive feature vector that combined standard statistical metrics with spectral and temporal descriptors, capturing subtle variations in signal dynamics:

- Statistical Moments: Mean, variance, skewness, and kurtosis to describe the amplitude distribution.
- Temporal Dynamics:
  – Slope: Linear trend of the signal, indicating overall rising or falling patterns.
  – Peak-to-Peak and Crest Factor: Measures of signal range and extreme values relative to the RMS level.
  – Zero-Crossing Rate (ZCR): Evaluated the oscillation frequency around the mean.
  – Asymmetry Index: A custom metric defined to quantify the imbalance between rising and falling slopes, crucial for distinguishing directionality (e.g., approaching vs. receding). It is calculated as the normalized difference between the energy of rising and falling slopes:
    (4) $$I_{asym}=\frac{\sum_{\Delta x_{i}>0}{\left(\Delta x_{i}\right)}^{2}-\sum_{\Delta x_{i}<0}{\left(\Delta x_{i}\right)}^{2}}{\sum_{i}{\left(\Delta x_{i}\right)}^{2}}$$
    where $\Delta x_{i}$ represents consecutive signal differences.
  – Autocorrelation (Lag-1): Measured the persistence of the signal values.
- Spectral and Time–Frequency Domains:
  – Spectral Centroid and Entropy: Extracted via Welch’s method to characterize the “center of mass” and complexity of the power spectrum.
  – Wavelet Energy: Energy of the detail coefficients at level 3 using the Daubechies 4 (db4) wavelet, capturing transient features localized in time and frequency.

These features were widely used in signal characterization [31,32,33,34].

#### 3.3.2. Feature Selection Strategy

Given the large number of extracted features, the selection strategy combined linear and non-linear analysis to identify the most relevant subset and avoid the curse of dimensionality:

1. Correlation Analysis: Pearson’s correlation coefficient measured the linear relationship between each feature and the target variables (Direction/Orientation), providing an initial interpretability check.
2. mRMR Selection (Final Criteria): The Minimum Redundancy Maximum Relevance (mRMR) algorithm [35] selected the final input features for clustering. Specifically, the implementation optimized a hybrid criterion combining non-linear relevance with linear redundancy control:
  (5) $$\mathrm{Score}\left(f_{i}\right)=I\left(f_{i};y\right)-\frac{1}{\left|S\right|}\sum_{f_{s}\in S}\left|\rho \left(f_{i},f_{s}\right)\right|$$
  where $I(f_{i};y)$ denotes the mutual information between the candidate feature $f_{i}$ and the target class *y* (Relevance), and $\left|\rho (f_{i},f_{s})\right|$ represents the absolute Pearson correlation between the candidate feature and the already selected features $f_{s}\in S$ (Redundancy). This approach ensured that the selected subset was highly predictive of the target while minimizing collinearity among features.

Based on this process, the top three most discriminative features were selected for each experimental setup to feed the clustering algorithms. Additionally, Principal Component Analysis (PCA) was applied to the scaled feature vectors to further reduce dimensionality and facilitate cluster visualization.

### 3.4. Normalization

To prevent scale-induced bias during training, all input features were normalized to a standard range before being fed into the classifiers. Given the distinct physical nature and dynamic ranges of the signals, RSSI (measured in dBm) and Phase (measured in degrees), normalization proceeded independently for each signal type. This prevented the variable with the larger numerical range (typically Phase) from dominating the gradients or distance metrics during training. A Min-Max scaling approach mapped values to the [0,1] interval, as described by:

(6) $$x_{scaled}=\frac{x-x_{min}}{x_{max}-x_{min}}$$

where *x* represents the original value, $x_{min}$ and $x_{max}$ correspond to the minimum and maximum values observed in the training set for that specific feature.

### 3.5. Modeling Approaches

To robustly estimate the movement direction and box orientation, the modeling strategy encompassed two main paradigms, namely unsupervised learning, to explore data separability without labels using clustering algorithms, and supervised learning, where predictive models were built using both classical classifiers and deep learning architectures operating on raw signal representations. Preliminary experiments with N=200 samples were conducted to determine the optimal hyperparameters and network architectures through a combination of grid search (for clustering and classical models) and empirical evaluation of architectural variants (for deep learning models), using peak 10-fold cross-validation accuracy as the selection criterion. To ensure reproducibility, a global random seed of 42 fixed all stochastic components, including model initialization, data splitting, and clustering algorithms.

#### 3.5.1. Unsupervised Learning

For exploratory analysis and pattern discovery, three clustering algorithms were evaluated: K-Means [36], Agglomerative Clustering (Agg) [37], and Mean Shift [38]. These algorithms assessed the intrinsic separability of the dataset based on the extracted feature set described in Section 3.3, allowing for the identification of patterns and the classification of information without the need for predefined labels [39]. The specific hyperparameters used for each clustering algorithm are detailed in Table 3. For K-Means and Agglomerative Clustering, the number of clusters *k* was set to 2 for direction detection and 3 for orientation estimation, matching the ground truth classes.

#### 3.5.2. Supervised Learning

In the supervised context, two distinct modeling approaches were compared: classical machine learning classifiers operating on flattened input vectors, and deep learning architectures operating directly on raw time-series signals.

##### Classical Machine Learning

Five discriminative algorithms were evaluated: Extra Trees [40], Random Forest [41], Support Vector Machine (SVM) [42], LightGBM (LGBM) [43], and XGBoost [44]. Each classifier received the normalized signal sequences flattened and concatenated into a single 1D feature vector $x_{ML}\in R^{2N}$, where *N* is the number of samples:

(7) $$x_{ML}=\left[RSSI_{1},\ldots ,RSSI_{N},\phi_{1},\ldots ,\phi_{N}\right]$$

The specific hyperparameters of the classical classifiers are summarized in Table 4.

##### Deep Learning and Hybrid Architectures

To leverage the sequential nature of RFID signals, deep learning models learned representations directly from raw time-series data [45]. These models included Convolutional Neural Networks (CNN) [46] and Residual Networks (ResNet) [47] for hierarchical feature extraction, and Long Short-Term Memory (LSTM) networks [48] for temporal dependency modeling. In addition, two hybrid architectures were included: CNN-LSTM, combining local feature extraction with temporal modeling [49], and the CNN+SVM model that replaced the standard softmax layer with a margin-based classifier to improve boundary separation [50]. For these models, the data was structured as a multi-channel time-series tensor $X_{DL}\in R^{N\times 2}$ to preserve temporal locality:

(8) $$X_{DL}=\left[\begin{array}{cc} RSSI_{1} & \phi_{1}\\ \vdots & \vdots \\ RSSI_{N} & \phi_{N} \end{array}\right]$$

All deep learning models ran on TensorFlow/Keras, with architectural details and training hyperparameters reported in Table 5.

### 3.6. Validation Strategy and Performance Metrics

Model performance was evaluated across both controlled and unstructured settings, using distinct validation strategies to isolate the specific domain shifts present in each environment.

#### 3.6.1. Laboratory Validation Protocol

For the controlled experiments, a stratified 10-fold cross-validation scheme [51] evaluated model generalization on the balanced dataset, preserving class distribution across all folds. Within each training partition, a further 20% subset served as a validation set for hyperparameter tuning and early stopping of the deep learning models.

As each tag pass was treated as an independent sample, the total dataset comprised 2400 samples for direction estimation (600 passes × 4 tags) and 7200 samples for orientation estimation (1800 passes × 4 tags).

#### 3.6.2. Shop Floor Validation Protocol

In the highly unstructured industrial shop floor scenario, a stratified 80/20 split first partitioned the dataset into training and test subsets, preserving class proportions. Each tag pass was treated as an independent sample, yielding total datasets of 2320 samples (4 tags × 2 directions × 290 passes) at 1 m and 1760 samples at 2 m (4 tags × 2 directions × 220 passes). To produce stable performance estimates from the limited test set (290 and 220 passes per direction at 1 m and 2 m, respectively), 1000 independent bootstrap [52] iterations were sampled with replacement from the test subset, providing confidence intervals that reflect the natural variability in operator speed, trajectory, and signal quality. Bootstrap resampling was preferred over fixed-partition schemes, which would yield inadequately small and potentially unrepresentative subsets for stable performance estimation. Furthermore, the analysis examined the influence of the gaps defined in Section 3.2.4 to evaluate robustness under different levels of signal fragmentation. Due to the focus on deployment feasibility, only the top-performing models identified in the laboratory experiments (Section 3.6.1) entered this real-world evaluation.

#### 3.6.3. Performance Metrics

Predictive performance was quantified through Accuracy (Acc), macro-averaged Precision (Pre), Recall (Rec), and F1-Score (F1) [53]. The macro-averaging strategy was deliberately selected to treat all classes equally, reflecting the balanced distribution of the dataset. Additionally, inference latency was recorded to determine operational feasibility for edge deployment.

### 3.7. Implementation Details

The experimental pipeline ran in Python 3.10.8, utilizing scikit-learn 1.3.0 for the classical algorithms and TensorFlow 2.10 for the deep neural networks. All training and inference ran on a Windows 11 workstation powered by an AMD Ryzen 9 5900HS CPU (Advanced Micro Devices, Inc., Santa Clara, CA, USA) (24 GB RAM) and an NVIDIA RTX 3060 GPU (NVIDIA Corporation, Santa Clara, CA, USA) (6 GB VRAM). To mitigate computational overhead during repeated training runs across folds and multiple resampling sizes *N*, all preprocessing and augmentation steps were executed offline. The resulting feature vectors were serialized to disk in tabular format consistent with Equation (7), with the target label appended as the final column. At loading time, the training loop reshaped them into task-specific input representations defined in Equations (7) and (8).

## 4. Results

This section presents a systematic evaluation of the proposed methodology, structured into three main experimental scenarios. First, Section 4.1 analyzes the performance of direction estimation in a controlled laboratory environment, including feature relevance assessment and model benchmarking. Second, Section 4.2 extends this analysis to the orientation classification task. Finally, Section 4.3 validates the system’s robustness in a real-world industrial shop floor setting, discussing the impact of environmental noise and signal fragmentation on model accuracy.

### 4.1. Direction Estimation

#### 4.1.1. Assessment of Signal Characteristics 

The feature selection output detailed in Table 6 shows that the linear trend (slope) of either RSSI or Phase was the primary predictor for direction, maintaining its top rank across all tested sample sizes (*N*). The dominant signal modality, however, shifted depending on the tag position. Tags 2 and 3 were mainly characterized by the *Slope_phase*, where correlations reached |R|=0.973. In contrast, Tags 1 and 4 depended on the *Slope_rssi* (|R|≈0.87). This positional dependence suggested that while Phase offered strong predictability for certain angles, RSSI acted as the main predictor when spatial geometry changed. Looking at the broader mRMR behavior, the algorithm built a complementary set of features. The primary feature consistently exhibited a strong linear correlation with the target (|R|>0.75, p<0.01), confirming that top-ranked selections were not driven by sample artifacts. However, secondary and tertiary features often displayed negligible linear correlations and correspondingly high *p*-values (e.g., |R|<0.05, p=0.56 for *ZCR_phase*). Since mRMR is based on Mutual Information, this selection pattern indicated that lower-ranked variables provided non-redundant information.

**Figure A1:**
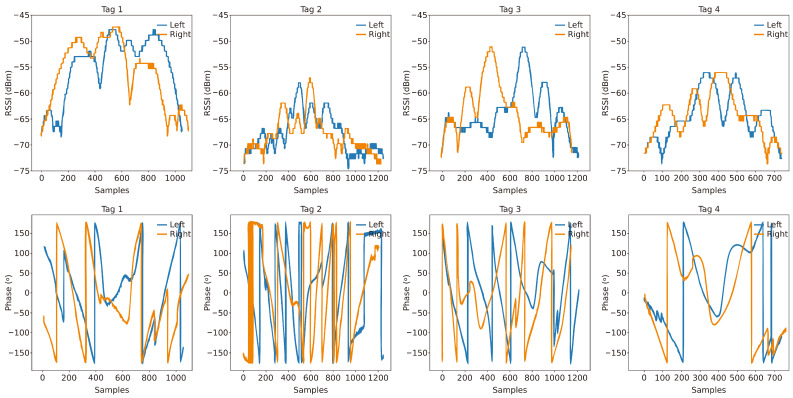
Representative profiles of raw RSSI (**top**) and Phase (**bottom**) signals acquired from Tags 1–4 during distinct directional movements (left: blue, right: orange), prior to the temporal resampling stage.

To support these mathematical rankings, the empirical raw signals are provided in Appendix A (Figure A1). The temporal plots showed clear physical signatures tied to the movement direction. Specifically, Phase profiles had noticeable rising and falling slopes for Tags 2 and 3, while RSSI shapes presented asymmetric envelopes. These visible morphological traits aligned with the features extracted and prioritized by the classification models.

#### 4.1.2. Unsupervised and Supervised Approaches

The direction classification accuracy for all evaluated models is presented in Table 7, showing how both sample size (*N*) and tag placement affect performance, with clear stability differences between unsupervised and supervised approaches. Macro-averaged Precision, Recall, F1-Score, and latency profiles are provided in Appendix B and Appendix C.

##### Unsupervised Clustering Performance

The clustering algorithms maintained consistent performance across Tags 1–3, with accuracies exceeding 96%, corroborating the feature analysis in Section 4.1.1. However, Tag 4 introduced higher variability: K-Means and Agglomerative Clustering sustained accuracies above 90%, whereas MeanShift collapsed to ≈64% at N=250 (standard deviation up to 22% across folds). This instability is attributed to the near-symmetric signal morphology of Tag 4, caused by its placement on the face opposite the antenna. Computationally, all unsupervised methods achieved inference latency below 8μs.

##### Supervised Learning Performance

Supervised models achieved the highest accuracy across all configurations. Classical machine learning ensembles (Extra Trees, Random Forest, SVM, LGBM, XGBoost) reached stable mean accuracies of 99.8%±0.5% standard deviation across almost all tested conditions. Among the deep learning architectures, only the hybrid CNN+SVM matched this baseline (>99.7%). The remaining neural network models (ResNet, CNN, LSTM and CNN-LSTM) showed greater fluctuation depending on the tag. For example, on Tag 1, mean accuracy decreased to 85% for the CNN-LSTM and 75% for the ResNet models. Regarding performance between directions, a difference of 0.1% was observed. In terms of processing time, the lowest peak execution was recorded by CNN+SVM (≈64 μs), followed by SVM and XGBoost (≈140 μs).

##### Sample Size Sensitivity

Evaluating the sample size (*N*), classical supervised models maintained their performance stability from N=50 up to N=300. This indicated that short data sequences contained the required metrics for accurate direction estimation, an important factor for the real-time evaluations detailed later in Section 4.3. Conversely, the unsupervised algorithms, particularly Mean Shift, showed higher sensitivity to variations in *N*, requiring different window sizes to establish stable decision boundaries.

### 4.2. Orientation Estimation

#### 4.2.1. Assessment of Signal Characteristics

Table 8 indicates that orientation was primarily characterized by descriptors in the dispersion and frequency domains, which is consistent with stronger multipath/NLoS sensitivity under rotation. Across almost all sample sizes for Tags 1, 2, and 4, *Var_phase* consistently dominated as the primary feature (|R|≈ 0.23–0.79), while Tag 3 predominantly depended on a combination of spectral and statistical RSSI descriptors, including *SC_rssi* (|R|≈0.37) and *Skew_rssi* (|R|≈0.67). The mRMR rankings further highlighted the non-linear nature of the orientation task. In contrast to direction, where the top-ranked features tended to exhibit high linear correlation (|R|>0.85), orientation often assigned rank-1 to features with modest Pearson coefficients (e.g., *WE3_rssi* for Tag 1 at N=50, R=0.186 and *SC_rssi* for Tag 4, R≈0.047). This pattern was compatible with non-monotonic class responses, for which Pearson’s *R* might understate dependence, while MI-based criteria could still prioritize variables that reduce class uncertainty. This complementarity was also visible in lower ranks. For Tag 1 at N=300, after selecting Phase variance, mRMR selected *SE_rssi* and *ZCR_phase*, combining spectral and temporal statistics across both modalities into a less redundant subset.

**Figure A2:**
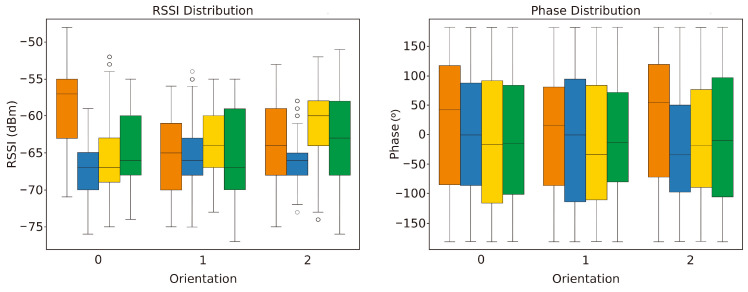
Variation of RSSI and Phase distributions across different box orientations without pre-processing (Orange: Tag 1; Blue: Tag 2; Yellow: Tag 3; Green: Tag 4).

The same effect was observable in the raw distributions reported in Appendix A (Figure A2). Phase values remained broadly spread over [−π,π], while RSSI showed orientation-dependent shifts in its interquartile range and occasional outliers (notably for Tags 2 and 3), supporting the emphasis on dispersion and spectral descriptors rather than purely trend-based features.

#### 4.2.2. Unsupervised and Supervised Approaches

Table 9 presents the classification accuracy for orientation estimation across all evaluated models. Relative to the binary direction task, the three-class orientation setting yielded lower separability for clustering methods, while supervised models preserved high accuracy across tags. Detailed macro-averaged classification metrics (Precision, Recall, and F1 score) and computational latency profiles are provided in Appendix B and Appendix C, respectively.

##### Unsupervised Clustering Performance

Clustering performance was less stable than in the direction task. Tags 1 and 2 remained high in most configurations (typically >94%), but specific settings degraded sharply. For example, for Tag 2 at N=50, K-Means dropped to 59%. Tag 3 exhibited the opposite pattern, where Mean Shift outperformed both K-Means and Agglomerative Clustering (>99% vs. ≈87%). Tag 4 remained the most challenging configuration, with clustering accuracies in the 72–80% range for N≥100. This average reflects a strong imbalance across orientations: while Orientation 1 was reliably classified (>99%), Orientations 0 and 2 both degraded to near-chance levels. In these two cases, NLoS blockage and polarization mismatch produce similar RSSI and phase profiles. Regarding inference times, unsupervised methods achieved very low latency (<2 μs), with the Agglomerative method achieving the lowest (≈0.07 μs).

##### Supervised Learning Performance

Supervised models achieved higher and more stable accuracies. Classical machine learning models maintained a mean accuracy above 99.6% across tags and sample sizes, and the deep learning/hybrid models (ResNet, CNN-LSTM, CNN+SVM) often achieved accuracy close to 100%. CNN and LSTM showed higher fold-to-fold variability, with standard deviations reaching 5.1% for Tag 4 and 9.3% for Tag 2, whereas CNN+SVM remained below 0.8%. Confusion matrices indicated that the few remaining errors were concentrated on Orientation 3 for Tag 4, where for the best-performing models, the accuracy remained above 99.5%. In terms of latency, CNN+SVM reached a peak inference time of ≈67 μs, close to LGBM (≈58μs), while deeper architectures were slower (e.g., CNN-LSTM at ≈4.2 ms).

##### Sample Size Sensitivity

Orientation clustering was sensitive to small windows, particularly at N=50. In contrast, supervised models maintained high performance even at N=50, indicating that short windows were sufficient for reliable orientation estimation under the supervised setting.

### 4.3. Industrial Shop Floor Evaluation

After the laboratory evaluation, the system was assessed in an unstructured industrial shop floor environment (Section 3.1.2), targeting direction estimation under higher kinematic variability and stronger multipath/NLoS effects than those observed in the controlled setup. Based on the laboratory results, the evaluation narrowed its focus to classical ML models (Extra Trees, Random Forest, SVM, LGBM, XGBoost) and the CNN+SVM hybrid.

The evaluation proceeded iteratively, beginning with a direct deployment test, which subsequently necessitated a structured adaptation strategy due to the results obtained. The experimental progression is summarized in Table 10.

As detailed in Table 10, the evaluation started with the zero-shot approach (EXP 1 and EXP 2), which directly tested models trained solely on laboratory data against shop floor acquisitions.

In response to the performance degradation observed in this initial test, EXP 3 introduced a domain adaptation step using exclusively the new unstructured 1 m data. To prevent negative transfer from the controlled laboratory datasets, classical machine learning algorithms were retrained from scratch. Conversely, for the hybrid CNN+SVM architecture, Transfer Learning (TL) was employed to fine-tune the previously learned representations. A stratified 80/20 split partitioned this dataset into training and test subsets, preserving class proportions. Data augmentation (Section 3.2.2) was applied exclusively to the training subset, with 20% reserved for hyperparameter tuning and early stopping. Final performance is reported on the hold-out test set using the predefined bootstrap protocol.

To assess spatial generalization, the EXP 3 models were evaluated on the independent 2 m dataset (EXP 4). Finally, to ensure maximum robustness for practical implementation, EXP 5 adopted the same partitioning and augmentation protocol as EXP 3, but with adaptation and testing performed on a merged dataset combining both the 1 m and 2 m acquisitions.

#### 4.3.1. Zero-Shot Evaluation (EXP 1 and EXP 2)

Applying laboratory-trained models directly to the shop floor data led to a marked reduction in performance. As reported in Table A4 of Appendix D, the 1 m zero-shot test (EXP 1) showed that several classical models retained moderate accuracy for Tag 1 (e.g., Random Forest and LGBM at ≈75–81%), whereas Tags 3 and 4 dropped towards chance-level performance (≈8–54%) across most classical architectures.

At 2 m (EXP 2), the degradation pattern changed: Tag 4 showed a modest recovery, while Tags 2 and 3 remained near ≈50% in multiple configurations. The CNN+SVM hybrid was also sensitive under zero-shot conditions, with accuracies ranging from ≈40% to 65% depending on tag and distance. Overall, these results indicated limited direct transfer from the controlled laboratory domain to the shop floor acquisitions without adaptation.

#### 4.3.2. Domain Adaptation and Cross-Distance Constraints (EXP 3 and EXP 4)

EXP 3 adapted the models using only the 1 m shop floor dataset (Appendix D, Table A5). After adaptation, classical ensembles (Extra Trees, Random Forest, LGBM, XGBoost) reached >96% accuracy for Tags 1, 3, and 4. Tag 2 improved to the 80–90% range for most classical models.

In EXP 4, the models adapted at 1 m were evaluated on the independent 2 m dataset. Performance decreased substantially in this cross-distance test; for Tags 1 and 3, accuracy approached ≈50% across multiple classical models (Appendix D, Table A5). This result highlighted a strong distance sensitivity when training and evaluation ranges differ.

#### 4.3.3. Unified Deployment Architecture (EXP 5)

To mitigate cross-distance sensitivity, EXP 5 utilized a unified dataset combining both 1 m and 2 m acquisitions, with overall performance summarized in Table 11. Furthermore, to ensure the unified model maintained balanced generalization and was not biased toward one specific spatial configuration, its performance was systematically evaluated on isolated 1 m and 2 m test sets (detailed in Table A6 of Appendix D).

The spatial unification effectively neutralized the distance-induced vulnerabilities. Under EXP 5, classical ensembles (Random Forest, Extra Trees, LGBM, XGBoost) achieved high and stable accuracy for Tags 1 and 4 (typically >97%). Tags 2 and 3 improved relative to the zero-shot setting, reaching ≈83–89% for several classical models. Despite its superiority in the controlled laboratory environment, CNN+SVM showed lower stability in this scenario, with Tag 2 ranging from ≈59–74% and Tag 1 from ≈74–81%, depending on *N*. Unlike end-to-end trained ensembles, CNN+SVM relies on a fixed feature representation learned under controlled conditions that degrades under the burst fragmentation and morphological variability of the shop floor environment. Detailed error analysis indicated that under zero-shot transfer (EXP 1 and EXP 2), directional discriminability degraded toward chance levels rather than exhibiting a systematic class skew, suggesting a general loss of morphological structure for Tags 2 and 3. Under unified domain adaptation (EXP 5), residual misclassifications revealed a consistent directional asymmetry, with leftward movement achieving higher accuracy than rightward movement across all tags and classical models (Table A7). For tags in geometrically favorable positions (Tags 1 and 4), this asymmetry remained below 2%, whereas for tags with oblique antenna-beam alignment (Tags 2 and 3), the asymmetry reached up to approximately 5% and 11%, respectively, suggesting that the dominant source of interclass imbalance is geometric rather than operational. Detailed per-class accuracy for EXP 5 across all tags, sample sizes, and models is provided in Table A7 of Appendix E. These results also validated the physics-informed data augmentation strategy, as when trained without DA, XGBoost accuracy for Tag 1 degraded to 66.8%, a reduction of ≈30 pp relative to the DA-trained counterpart (97.1%), confirming that domain-invariant augmentation was a necessary condition for cross-domain generalization even for classical ensemble methods.

Regarding signal fragmentation (Section 3.2.4), Table 12 shows a consistent association between higher gap severity and lower accuracy. For structurally favorable configurations, Tags 1 and 4, Extra Trees and XGBoost exhibited limited degradation (e.g., ≈4–5% from Level 0 to Level 3). Intriguingly, these two models displayed counterintuitive behavior for Tag 1, demonstrating slightly higher performance on fragmented samples (Levels 1 to 3) compared to clean signals. For Tag 3, the degradation was larger, with drops up to ≈20–27% at Level 3 relative to the corresponding Level 0 baselines, with Level 3 representing 19% of Tag 3 passes, the highest proportion observed across all tag positions. This tag was positioned closer to the pallet rack handle and the operator, making it more susceptible to operator-induced NLoS obstruction and signal shadowing, which constitutes the primary physical mechanism underlying its disproportionate loss of accuracy under severe fragmentation. Window size also affected robustness: N=50 was the most sensitive to gaps, whereas larger windows (N≥150) reduced the fragmentation penalty.

Inference latency remained below 1 ms for the evaluated classical ensembles. LGBM showed the lowest absolute peak (≈10 μs), followed by Extra Trees (≈136 μs) and XGBoost (≈150 μs). Complete latency profiles are provided in Appendix C.

Collectively, these results provide a multi-variable sensitivity characterisation of the proposed system, spanning distance (evaluated under zero-shot and cross-distance conditions, Section 4.3.1 and Section 4.3.2), window size (Section 4.1 and Section 4.2), tag position, and fragmentation level, while environmental geometry is identified as a limitation (Section 5).

## 5. Discussion

With the advancements of Industry 4.0, RFID technology has been widely adopted across various industrial sectors, particularly in logistics and manufacturing process control, which require systems capable of extracting spatiotemporal information from highly unstructured environments. In contrast to approaches that assume stable radio conditions, this study explicitly evaluated both controlled laboratory acquisitions and an unstructured shop floor deployment, including distance shifts, human-induced kinematic variability, and signal fragmentation.

The laboratory evaluation revealed that direction and orientation estimation present fundamentally different challenges. For direction estimation, the tag’s physical translation relative to the antenna produces a highly monotonic signal variation. Feature analysis supported this interpretation, showing that simple linear trend descriptors, the slope of either RSSI or Phase, consistently dominated as predictors. Interestingly, the optimal mode (Phase vs. RSSI) depended entirely on tag placement. This is consistent with the geometry-driven nature of RF propagation, as small spatial changes determine whether phase stability or amplitude attenuation carries the most reliable discriminative structure. The model comparison reinforced this interpretation. Unsupervised clustering reached high accuracy for several tags (>97%), which suggests that the engineered features encode direction in a way that is, in many configurations, naturally separable even without labels. However, the Mean Shift degradation on Tag 4 highlighted that clustering robustness is not uniform and depends on how well algorithmic assumptions match the feature distribution. Regarding supervised learning, classical ensembles (Extra Trees, Random Forest, LGBM, XGBoost) and SVM-based models, including the hybrid CNN+SVM architecture, demonstrated the ability to accurately determine movement direction regardless of tag placement. These models consistently achieved near-perfect accuracies (≈100%), showing negligible sensitivity to window size and confirming that even short signal sequences captured the essential directional dynamics (performance variations below 1%). Each sample corresponds to a single unidirectional pass per tag, representing an independent observation with a morphologically distinct signal profile, with the global random seed ensuring pre-fold shuffling and precluding ordering bias from the sequential acquisition structure. With 100 repetitions per speed–direction combination, the systematic signal variation across the 2800 mm conveyor belt provided sufficient discriminability for near-perfect classification even with DA, suggesting that the signal structure is inherently discriminative under controlled conditions. These results further suggested that highly complex architectures such as ResNet are not necessary for optimal performance in this binary task under controlled conditions. Their added computational capacity does not translate into practical gains when the discriminative structure is already well captured by compact features and robust decision boundaries.

Orientation estimation, in contrast, proved more challenging than direction because it involves three classes and the RF response to rotation is rarely monotonic. This behavior was reflected in the mRMR rankings, which favored dispersion metrics (phase variance) and spectral descriptors (RSSI spectral centroid), and occasionally assigned rank-1 to features with low Pearson correlation (|R|≈ 0.04–0.38). This divergence is consistent with non-monotonic class responses, where mutual-information criteria can capture dependencies that linear correlation underestimates. As a result, unsupervised methods were less stable: performance dropped sharply in specific settings (e.g., Tag 2 with N=50), suggesting that very short windows may be insufficient to form reliable multi-pose centroids for some tags. Conversely, the inversion observed for Tag 3 (Mean Shift outperforming K-Means/Agglomerative) indicated that, depending on placement, the class structure can align better with density-based separation than with spherical centroid assumptions. Supervised models, however, mitigated these limitations. The highest accuracies were achieved by the ResNet architecture, followed by the hybrid CNN+SVM model and the classical ensembles, consistently exceeding 99.5% in most configurations, while CNN and LSTM exhibited occasional degradations (down to ≈91% and ≈97% in specific cases). These localized failures are consistent with stronger multipath and NLoS perturbations during rotation. The metallic components inside the case (iron and aluminum) likely increased reflections and attenuation, and while circular polarization mitigates these effects, it cannot completely eliminate them. Overall, the high accuracy values reflected the intrinsic discriminability of the controlled setup, with stratified cross-validation and fixed-seed shuffling ensuring that no ordering artifacts influenced fold composition. For orientation estimation, the positioning geometry of the tags drove the primary variability, given that NLoS occlusion caused by the rotation introduced performance differences between tag placements of less than 3% for the best-performing models and up to 9% for the LSTM architecture, while window size and model complexity were secondary factors. Classical ensembles performed on par with deep architectures (<0.5%), suggesting that tag placement may have a larger impact on robustness than model complexity.

**Figure 4:**
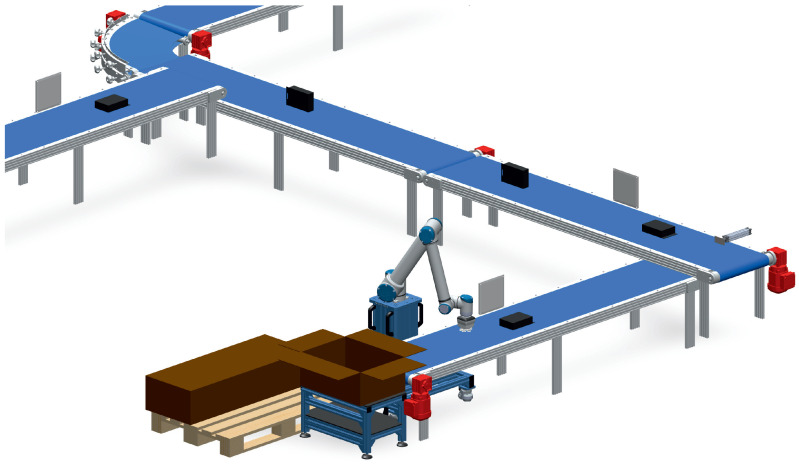
Conceptual implementation of the single-antenna RFID orientation system integrated with automated robotic palletization and logistics sorting.

Overcoming these algorithmic challenges to reliably estimate RFID tag orientation presents a significant opportunity for modern industrial logistics. While binary direction tracking confirms the general flow of goods, spatial orientation dictates handling mechanics. Figure 4 illustrates a practical deployment scenario where orientation inference drives automated transportation and distribution. By ensuring that items pass through validation checkpoints in the correct alignment, the proposed single-antenna system can directly inform downstream robotic handlers. This capability optimizes robotic palletization, increases storage density, and minimizes mechanical errors during automated sorting, simultaneously ensuring that the physical product flow has occurred without unauthorized or accidental spatial disturbance.

The transition from the controlled conveyor to the industrial shop floor exposed a substantial domain gap. Zero-shot transfer evaluation of the best-performing models, which were trained exclusively on laboratory data and tested directly on organic shop floor acquisitions, revealed a substantial degradation in accuracy, and, for several tags, performance close to chance level. This confirmed that the motion signals learned under clean, repeatable kinematics do not directly generalize to trajectories subject to human handling, pallet-jack dynamics, and background activity. Domain adaptation with organic 1 m data (EXP 3) largely restored performance for classical ensembles, which again achieved high accuracies on several tags (>97%). Nevertheless, the cross-distance evaluation (EXP 4) revealed a strong distance sensitivity: models optimized at 1 m often failed when applied to 2 m data, with accuracy dropping towards 50% for some tags. In practice, this indicates that range acts as an implicit confounder, modifying the relationship between features and labels unless multiple distances are represented during training. The unified training configuration (EXP 5), where 1 m and 2 m datasets were merged under a consistent adaptation protocol, mitigated these effects and yielded the most stable shop floor behavior, suggesting that explicitly modeling distance variation at training time is important for robust deployment. These results confirm that the proposed system requires target-domain calibration data prior to deployment. Under unified shop floor conditions, the best model in the controlled tests, the CNN+SVM hybrid, displayed lower stability, with accuracy ranges as low as 59–74% on some tags, whereas Random Forest, Extra Trees, LGBM and XGBoost consistently maintained accuracies above 97% for favorable geometries and above 83% for the more difficult NLoS configurations.

The analysis of signal fragmentation added an important operational dimension. For tags in structurally favorable positions (e.g., Tag 1), classical ensembles like XGBoost demonstrated notable resilience, sometimes performing comparably or better under moderate fragmentation. This counterintuitive behavior is consistent with the training distribution, as clean continuous signals (Level 0) can accumulate low-frequency drift and multipath variability across the full pass length, whereas moderately fragmented signals (Level 1–2) retain only the most stable burst segments, which more closely resemble the DA-augmented training samples. This pattern was consistent across both antenna distances, reinforcing its interpretation as a systematic effect of the DA-augmented training distribution rather than a sampling artefact. In contrast, for Tag 3, mounted perpendicularly to the antenna, fragmentation progressively degraded performance from Level 0 to Level 3, as its oblique signal geometry makes directional inference more sensitive to data loss. Moreover, short windows (N=50) were consistently the most sensitive to gaps, whereas longer windows (N≥150) attenuated the impact of bursty reads. These results suggest two complementary levers for practitioners: choosing window sizes that balance latency and robustness, and designing tag placement strategies that minimize systematic occlusion along likely trajectories. From an IIoT perspective, computational constraints reinforced this recommendation. The latency analysis showed that gradient-boosted and tree-ensemble models achieve peak inference times in the microsecond range, with LGBM at approximately 10 μs, Extra Trees at roughly 136 μs, and XGBoost around 150 μs. All classical models remained below the 1 ms threshold across window sizes, which is compatible with high-speed conveyor operation on resource-constrained embedded devices. This combination of robustness after adaptation and very low computational cost positions these ensembles as viable candidates for edge deployment in real warehouses and distribution centers. Across the shop floor variables evaluated, antenna-to-tag distance was the dominant sensitivity factor, collapsing accuracy to ≈50% under cross-distance transfer and restored to >97% with unified multi-distance training (EXP 5). Under unified deployment, tag placement geometry was the most persistent source of variance (Tags 2 and 3 stabilized at 83–89% vs. >97% for Tags 1 and 4, reflecting geometrically unfavorable antenna-beam alignment). Signal fragmentation ranked third, with drops of up to 20–27% from Level 0 to Level 3 (Table 12). Window size had the smallest impact, with *N* = 50 most sensitive to gaps and N≥150 consistently reducing the fragmentation penalty.

Beyond model-level inference costs, the complete preprocessing pipeline, comprising Savitzky–Golay smoothing, phase unwrapping, and cubic spline interpolation, contributed an average overhead of 0.94 ms per signal in laboratory conditions and up to 1.22 ms on the shop floor, with worst-case peaks below 20 ms. Alternative strategies with automated parameter selection, such as Generalized Cross-Validation (GCV) smoothing splines, were evaluated but introduced average overheads exceeding 25 ms with isolated peaks above 1 s, without measurable gain in classification accuracy. The adopted pipeline therefore minimized latency while fully preserving the morphological fidelity required for high predictive accuracy.

Taken together, these results address the three gaps identified in Section 2.4. The proposed single-antenna pipeline eliminates multi-antenna infrastructure, directly reducing hardware cost and deployment complexity. The performance gain over prior short-segment approaches [13,22] is attributable to the convergence of three design decisions: the reconstruction of the complete RFID pass, which preserves the approach-and-recede dynamics that short windows cannot capture; phase unwrapping prior to interpolation, which eliminates 2π discontinuities that degrade feature quality under FFT-based resampling; and task-calibrated augmentation intensity, which exposes models to the specific degradation modes encountered at deployment without masking the morphological signatures that carry discriminative information. However, direct numerical comparison remains approximate, given that the benchmarks of 75–92% were obtained without domain adaptation or exposure in a production environment. The observed improvement therefore reflects these design decisions in conjunction with the availability of target-domain training samples, not architectural superiority alone. The shop floor validation under distance variation, human-induced kinematics, and signal fragmentation provides the real-world robustness evidence absent from existing RFID-AI literature. Nevertheless, orientation estimation was not validated on the shop floor, and the transferability of physics-informed domain adaptation to this task under industrial conditions remains to be established in future work.

Beyond this, the experiments revealed current limitations, namely that the shop floor data was collected at only two discrete distances in a single industrial environment, and that signal-level vulnerability to severe NLoS occlusions and uncalibrated antenna-tag ranges was observed as a recurring source of degradation. Additionally, the antenna was maintained in a fixed configuration throughout (1 m height, perpendicular to the movement axis), leaving the system’s sensitivity to geometric parameters such as elevation angle, lateral offset, and approach angle unquantified. To address these limitations, three operational solutions are recommended:

1. Strategic Tag Placement: In operations involving pallet jacks or forklifts, tags should be positioned on the face with direct line-of-sight to the antenna, as NLoS configurations were the primary source of performance degradation across tag placements.
2. Optimization of Antenna Geometry: Future studies should investigate antenna placement configurations, specifically height and tilt angle, as the fragmentation analysis indicated that oblique read geometries amplify burst-mode reads, suggesting that optimized elevation could extend the effective read zone and reduce fragmentation onset without altering the software architecture.
3. Redundant Hardware as a Fallback: While the goal of this AI-driven approach is to minimize infrastructure, for extremely hostile RF environments (when signal fragmentation reaches severe levels or accuracy drops below acceptable operational margins) or critical entry points where tags are not yet registered in the database, the implementation of multi-antenna tunnels or secondary read points on the opposite side of the gantry may be necessary [54,55]. This ensures mandatory tag acquisition prior to system integration when AI alone cannot recover severely degraded signals.

Overall, the findings of this study provide the industrial logistics market with a practical strategy for continuous movement and orientation tracking using standard, off-the-shelf RFID hardware. Unlike classical DoA/AoA algorithms, which require multi-antenna arrays and carry $O(M^{3})$ eigendecomposition complexity, or BLE antenna-switching solutions, which rely on active tags and 2.4 GHz infrastructure incompatible with passive UHF RFID deployments, the proposed approach extracts directional and orientational intelligence directly from the temporal morphology of signals on existing single-antenna infrastructure, without hardware modification or calibration overhead. More broadly, the pipeline can be adapted to any industrial checkpoint equipped with a single passive UHF RFID antenna, provided that items move along conveyor belts, gantries, or dock-door systems and that tags remain readable during transit. Contexts involving omni-directional movement, highly variable approach trajectories, or metallic shielding that prevents consistent tag readability fall outside the scope of the current validation and would require dedicated adaptation studies. The application of carefully selected machine learning algorithms, specifically classical tree-based ensembles, improves spatial detection accuracy and operational efficiency while reducing the reliance on costly, complex multi-antenna infrastructures.

## 6. Conclusions

This study evaluated the feasibility of a single-antenna UHF RFID system, enhanced by machine learning, for estimating the movement direction and spatial orientation of logistics assets, demonstrating that accurate inference is achievable without multi-antenna infrastructure under the conditions tested.

For orientation estimation, laboratory results confirmed that the task is intrinsically more challenging than direction detection, yet all supervised models achieved near-perfect multi-pose classification (>99.5%). Although ResNet marginally outperformed classical ensembles, the performance gap was negligible (<0.5%) and outweighed by its computational overhead, ≈1500 μs compared to 150 μs for XGBoost, making classical tree-based ensembles the most practical choice for resource-constrained industrial deployment.

For direction estimation, the shop floor evaluation revealed a significant domain gap caused by distance variation and human-induced kinematics, which deep learning models could not overcome without adaptation. Following unified domain adaptation, XGBoost restored directional tracking accuracy to >97% for line-of-sight configurations. Despite these results, AI alone cannot fully compensate for adverse RF geometries, as extreme data loss combined with multipath interference degrades predictive power in NLoS configurations. Algorithmic robustness must therefore be coupled with strategic tag placement to avoid critical NLoS occlusions.

Together, these results address the three research objectives formulated in the introduction, generating three empirically grounded contributions to the state of the art in single-antenna RFID-AI systems. First, complete-pass RSSI and phase morphology encoded sufficient directional and orientational information for near-perfect supervised classification under controlled conditions (>99.5% accuracy across all supervised architectures), confirming that the proposed resampling strategy preserves the discriminative morphological structure of variable-length RFID passes and overcomes the 75–92% accuracy ceiling reported in prior single-antenna laboratory studies. Second, physics-informed data augmentation combined with target-domain adaptation proved important for cross-domain generalization. Without domain adaptation, accuracy dropped approximately 30 pp under shop floor conditions; with unified shop floor adaptation, XGBoost recovered direction accuracy to >97%, demonstrating that physically grounded augmentation can effectively bridge this laboratory-to-industrial gap, an approach that no prior single-antenna RFID-AI study has implemented or evaluated. Third, the systematic comparison of thirteen architectures demonstrated that classical tree-based ensembles match or exceed deep learning models within a margin of less than 0.5 pp, while operating at one to two orders of magnitude lower inference latency (XGBoost: ≈150 μs vs. CNN-LSTM: ≈4.2 ms), establishing classical ensembles as the recommended paradigm for resource-constrained IIoT edge deployment.

Future work will prioritize extending the orientation pipeline to shop floor environments, directly addressing the real-world validation gap identified for orientation estimation in Section 2.4. Antenna geometry sensitivity, which remains unquantified in the present study, along with performance under omni-directional movement trajectories, represents the primary open questions for broadening the generalization scope and will be investigated across diverse industrial environments and uncalibrated spatial configurations. By demonstrating that a single commercial UHF antenna, augmented with physics-informed machine learning, can approach the accuracy levels reported for more complex multi-antenna systems in controlled settings, this work provides an experimental basis for further investigation of software-defined sensing approaches in resource-constrained IIoT logistics deployments.

## Figures and Tables

**Table 1:** Physics-informed data augmentation parameters per noise scenario. Where parameters differ between tasks, values are reported as Direction/Orientation. Bounds ($\sigma_{RSSI}$) were calibrated from task-specific quartile distributions.

Parameter	A (Clean)	B (Moderate)	C (Noisy)	D (Extreme)
$\sigma_{RSSI}$ bounds (dB)	<3.35/<4.41	[3.35, 3.72)/[4.14, 5.15)	[3.72, 3.80)/[5.15, 5.36)	≥3.80/≥5.36
Dataset share (%)	13.7/24.6	62.7/25.9	13.3/24.4	10.3/25.1
Beta α	2.0	1.5	1.0	2.5
Distance range $d_{max}$ (cm)	±3	±8/6	±12/10	±2/14
Horiz. shift (%)	±3	±5	±10	±1/8
RSSI noise $\sigma_{r}$ (dB)	1.5/1.0	1.0/1.3	1.5/1.7	2.0
Phase noise $\sigma_{\phi }$ (°)	8/7	10/9	12/11	15/14
Window slicing $p_{w}$	0.25/0.15	0.35/0.25	0.40/0.35	0.35/0.40

**Table 2:** Modified Data Augmentation parameters for the unstructured shop floor domain. Additive Gaussian noise is disabled to preserve organic signal features.

Parameter	Very Mild	Mild	Moderate
Beta α	4.5–5.5	4.0–5.0	3.5–4.5
Vert. Shift ($p_{v}$)	0.10	0.30	0.50
Interference Gain (dB)	±0.1	±0.2	±0.3
Distance range (cm)	±1.0	±1.5	±2.0
Horiz. Shift ($p_{h}$)	0.20	0.20	0.30
Horiz. Shift (%)	0.5–1.0%	1.0–2.0%	1.0–3.0%
Window Slicing ($p_{w}$)	0.0 (None)	0.20	0.50
Window Removal (%)	-	2–5%	5–10%

Micro-thermal noise (σ=0.2 dBm) is exclusively applied at the reader’s sensitivity boundary (−85 dBm) to prevent artificial linearity.

**Table 3:** Optimized hyperparameters for the clustering algorithms. Where parameters differ between tasks, values are reported as Direction/Orientation.

Parameters	K-Means	Agg	Mean Shift
N° clusters	2/3	2/3	–
Initialization	random	–	–
n_init	10/5	–	–
Eps/Bandwidth	–	–	0.1774/0.1241
Min Samples	–	–	–
Linkage	–	ward	–
Bin Seeding	–	–	True

**Table 4:** Optimized hyperparameters for the classical classifiers. Where hyperparameters differ between tasks, values are reported as Direction/Orientation.

Parameters	Extra Trees	Random Forest	SVM	LGBM	XGBoost
N° Estimators	300/100	500/300	–	100/300	500/300
Max Depth	10/20	10	–	5	5/7
Bootstrap	True	–	–	–	–
Learning Rate	–	–	–	0.1	0.05
Kernel	–	–	rbf	–	–
C (Regul.)	–	–	1/10	–	–
Gamma	–	–	scale	–	–
Probability	–	–	True	–	–
Subsample	–	–	–	0.8/1.0	0.8

**Table 5:** Architectural configuration of the Deep Learning models. Where hyperparameters differ between tasks, values are reported as Direction/Orientation.

Model	Layer Structure (Sequential)	Training Configuration
**ResNet**	1. Init: Conv1D (32, k = 5) + BN + ReLU 2. ResBlock1: 2× Conv1D(32, k = 5), Add 3. ResBlock2: 2× Conv1D(64, k = 5), 1 × 1 Proj, Add 4. MaxPool + GlobalAvg + Dense(64, Drop 0.3)	**Optimizer:** Adam **Loss:** Cat. Cross-Entropy **Batch Size:** 32 **Epochs:** 50 (Early Stop)
**CNN**	1. Conv1D (64 filters, kernel = 7/5) + BN + ReLU 2. MaxPool1D (size = 2) 3. GlobalAvgPool1D 4. Dense (64, ReLU) → Softmax	
**LSTM**	1. BatchNormalization 2. LSTM (128 units, dropout = 0.3) 3. Dense (Softmax)	
**CNN-LSTM**	1. Conv1D (32/64 filters, k = 5) + BN + ReLU + MaxPool 2. LSTM (64/128 units, dropout = 0.2) 3. Dense (64, ReLU) → Softmax	
**CNN+SVM**	Extractor: Trained CNN (minus Softmax) Classifier: SVM (C = 1/10, rbf, gamma=scale)	

**Table 6:** Top-3 discriminative features selected by the mRMR algorithm for direction estimation across different tags and sample sizes. The Pearson correlation coefficient (*R*) and its 95% Confidence Interval (CI) are reported for each feature.

Tag	Samples	Ranking 1			Ranking 2			Ranking 3		
		Feature	R	CI (95%)	Feature	R	CI (95%)	Feature	R	CI (95%)
1	50	Slope_rssi	−0.862	[−0.873, −0.851]	P2P_rssi	0.078	[0.036, 0.119]	ZCR_phase	−0.009	[−0.051, 0.033]
	100	Slope_rssi	−0.871	[−0.881, −0.860]	WE3_rssi	0.028	[−0.014, 0.070]	WE2_phase	0.020	[−0.022, 0.062]
	150	Slope_rssi	−0.873	[−0.883, −0.863]	WE0_rssi	0.045	[0.003, 0.087]	Kurt_rssi	−0.217	[−0.257, −0.177]
	200	Slope_rssi	−0.875	[−0.884, −0.864]	SC_rssi	−0.388	[−0.424, −0.352]	P2P_rssi	0.077	[0.035, 0.119]
	250	Slope_rssi	−0.875	[−0.885, −0.865]	P2P_rssi	0.077	[0.035, 0.119]	SC_rssi	−0.394	[−0.429, −0.358]
	300	Slope_rssi	−0.876	[−0.885, −0.865]	P2P_rssi	0.077	[0.035, 0.119]	SE_rssi	−0.140	[−0.181, −0.098]
2	50	Slope_phase	0.816	[0.802, 0.830]	CF_phase	0.257	[0.218, 0.296]	Kurt_rssi	0.431	[0.396, 0.465]
	100	Slope_phase	0.884	[0.874, 0.892]	CF_phase	0.180	[0.139, 0.221]	Mean_phase	0.819	[0.805, 0.833]
	150	Slope_phase	0.887	[0.877, 0.895]	CF_phase	0.143	[0.101, 0.184]	Mean_phase	0.826	[0.812, 0.839]
	200	Slope_phase	0.888	[0.879, 0.896]	CF_phase	0.145	[0.103, 0.186]	Mean_phase	0.824	[0.810, 0.837]
	250	Slope_phase	0.889	[0.879, 0.897]	CF_phase	0.123	[0.081, 0.165]	Mean_phase	0.822	[0.808, 0.836]
	300	Slope_phase	0.889	[0.880, 0.897]	CF_phase	0.114	[0.072, 0.155]	Mean_phase	0.820	[0.806, 0.834]
3	50	Slope_phase	0.973	[0.970, 0.975]	Mean_phase	0.862	[0.851, 0.873]	Skew_rssi	−0.038	[−0.080, 0.004]
	100	Slope_phase	0.970	[0.968, 0.973]	Mean_phase	0.845	[0.832, 0.856]	WE3_rssi	−0.019	[−0.061, 0.023]
	150	Slope_phase	0.969	[0.967, 0.972]	Slope_rssi	−0.810	[−0.824, −0.795]	WE0_rssi	−0.023	[−0.065, 0.019]
	200	Slope_phase	0.969	[0.966, 0.971]	Slope_rssi	−0.811	[−0.825, −0.796]	CF_rssi	0.039	[−0.003, 0.081]
	250	Slope_phase	0.969	[0.966, 0.971]	Slope_rssi	−0.812	[−0.826, −0.797]	WE2_rssi	−0.001	[−0.043, 0.041]
	300	Slope_phase	0.969	[0.966, 0.971]	Slope_rssi	−0.813	[−0.827, −0.798]	P2P_rssi	0.028	[−0.015, 0.070]
4	50	Slope_rssi	−0.748	[−0.766, −0.729]	Asym_rssi	0.457	[0.423, 0.489]	CF_rssi	−0.073	[−0.115, −0.031]
	100	Slope_rssi	−0.758	[−0.775, −0.739]	Asym_rssi	0.439	[0.405, 0.473]	Kurt_rssi	0.086	[0.044, 0.128]
	150	Slope_rssi	−0.761	[−0.778, −0.743]	Asym_rssi	0.433	[0.398, 0.466]	SE_rssi	0.025	[−0.018, 0.067]
	200	Slope_rssi	−0.762	[−0.779, −0.744]	Asym_rssi	0.428	[0.393, 0.462]	P2P_rssi	−0.040	[−0.082, 0.003]
	250	Slope_rssi	−0.763	[−0.780, −0.745]	Asym_rssi	0.425	[0.390, 0.459]	Skew_phase	0.024	[−0.018, 0.066]
	300	Slope_rssi	−0.764	[−0.781, −0.746]	Asym_rssi	0.422	[0.387, 0.456]	Skew_phase	0.022	[−0.020, 0.064]

**Feature abbreviations:** Skew: Skewness; Kurt: Kurtosis; P2P: Peak-to-Peak; CF: Crest Factor; ZCR: Zero-Crossing Rate; Asym: Asymmetry Index; SC: Spectral Centroid; SE: Spectral Entropy; WE0: Wavelet Energy Level 0; WE2: Wavelet Energy Level 2; WE3: Wavelet Energy Level 3. Subscripts _rssi and _phase indicate the signal modality.

**Table 7:** Classification accuracy (%) for direction estimation across different tags and sample sizes (*N*). Values represent the mean accuracy obtained from stratified 10-fold cross-validation.

Tag	Samples	Clustering			Classical ML					DL and Hybrid Architectures				
		K- Means	Agg	Mean Shift	Extra Trees	Random Forest	SVM	LGBM	XGBoost	ResNet	CNN	LSTM	CNN- LSTM	CNN+ SVM
1	50	97.00	96.00	96.67	99.83	100.00	99.67	100.00	99.83	75.00	100.00	99.67	100.00	100.00
	100	96.83	96.17	96.50	99.83	100.00	99.67	99.83	99.83	75.00	100.00	100.00	100.00	100.00
	150	96.67	97.00	97.00	100.00	100.00	99.67	99.83	99.83	85.00	100.00	99.67	100.00	100.00
	200	97.67	96.33	97.17	100.00	100.00	99.67	99.83	99.83	90.00	100.00	99.83	96.33	100.00
	250	97.83	96.83	97.33	100.00	100.00	99.83	99.83	99.83	90.00	100.00	99.17	94.00	100.00
	300	96.33	96.83	97.00	100.00	100.00	99.67	99.83	99.83	85.00	99.83	99.17	85.00	100.00
2	50	91.83	93.33	93.00	99.83	99.83	100.00	100.00	99.83	81.17	97.00	100.00	100.00	100.00
	100	97.50	96.17	96.67	100.00	99.83	100.00	100.00	99.83	90.00	97.17	100.00	100.00	100.00
	150	98.67	96.33	97.00	99.83	99.83	100.00	99.83	99.83	87.50	96.00	99.83	100.00	100.00
	200	98.50	96.67	97.17	99.83	99.83	100.00	100.00	99.83	89.67	91.83	97.67	100.00	100.00
	250	98.67	96.67	98.00	99.83	99.83	100.00	99.83	99.83	88.50	94.83	98.50	100.00	100.00
	300	98.83	96.83	97.67	99.83	99.83	100.00	99.83	99.83	99.00	93.50	92.83	100.00	100.00
3	50	100.00	100.00	100.00	100.00	100.00	100.00	100.00	100.00	95.00	100.00	100.00	100.00	100.00
	100	100.00	100.00	100.00	100.00	100.00	100.00	100.00	100.00	95.00	100.00	100.00	96.67	100.00
	150	100.00	100.00	100.00	100.00	100.00	100.00	100.00	100.00	100.00	100.00	95.83	100.00	100.00
	200	100.00	100.00	100.00	100.00	100.00	100.00	100.00	100.00	90.00	100.00	99.67	100.00	100.00
	250	100.00	100.00	100.00	100.00	100.00	100.00	100.00	100.00	90.00	100.00	96.00	100.00	100.00
	300	100.00	100.00	100.00	100.00	100.00	100.00	100.00	100.00	100.00	90.00	93.17	100.00	100.00
4	50	95.00	90.17	68.50	100.00	100.00	100.00	100.00	100.00	90.00	99.83	100.00	100.00	100.00
	100	89.67	89.67	69.17	100.00	100.00	100.00	99.67	100.00	85.67	97.50	99.83	100.00	100.00
	150	89.83	81.67	75.00	100.00	100.00	100.00	99.83	100.00	92.00	99.83	99.83	100.00	99.81
	200	99.17	97.00	73.00	100.00	100.00	100.00	99.67	100.00	90.00	91.83	99.50	95.00	99.72
	250	99.17	99.33	64.67	100.00	100.00	99.83	99.83	100.00	80.00	93.50	99.50	100.00	99.72
	300	99.17	94.33	70.67	100.00	100.00	100.00	99.83	100.00	89.33	97.50	98.83	99.83	99.72

**Table 8:** Top-3 discriminative features selected by the mRMR algorithm for orientation estimation across different tags and sample sizes. The Pearson correlation coefficient (*R*) and its 95% Confidence Interval (CI) are reported for each feature.

Tag	Samples	Ranking 1			Ranking 2			Ranking 3		
		Feature	R	CI (95%)	Feature	R	CI (95%)	Feature	R	CI (95%)
1	50	WE3_rssi	0.186	[0.163, 0.210]	P2P_phase	0.525	[0.508, 0.543]	Mean_rssi	−0.726	[−0.737, −0.714]
	100	ZCR_phase	−0.257	[−0.279, −0.234]	Var_phase	0.690	[0.677, 0.702]	SE_rssi	−0.301	[−0.323, −0.279]
	150	Var_phase	0.692	[0.679, 0.704]	SC_rssi	−0.346	[−0.367, −0.324]	ZCR_phase	−0.346	[−0.367, −0.324]
	200	Var_phase	0.692	[0.679, 0.705]	SC_rssi	−0.350	[−0.371, −0.328]	ZCR_phase	−0.395	[−0.415, −0.374]
	250	Var_phase	0.691	[0.678, 0.704]	SC_rssi	−0.351	[−0.372, −0.330]	SE_phase	−0.315	[−0.336, −0.293]
	300	Var_phase	0.691	[0.678, 0.704]	SE_rssi	−0.323	[−0.345, −0.301]	ZCR_phase	−0.458	[−0.477, −0.439]
2	50	Var_phase	−0.342	[−0.363, −0.320]	ACF1_rssi	0.417	[0.397, 0.437]	Slope_phase	0.019	[−0.005, 0.043]
	100	Var_phase	−0.285	[−0.307, −0.262]	P2P_phase	−0.595	[−0.610, −0.579]	ACF1_rssi	0.373	[0.351, 0.393]
	150	Var_phase	−0.277	[−0.300, −0.255]	P2P_phase	−0.590	[−0.606, −0.574]	ACF1_rssi	0.347	[0.325, 0.368]
	200	Var_phase	−0.278	[−0.300, −0.255]	P2P_phase	−0.589	[−0.605, −0.573]	ACF1_rssi	0.332	[0.310, 0.353]
	250	Var_phase	−0.279	[−0.302, −0.257]	WE3_phase	−0.576	[−0.592, −0.559]	P2P_phase	−0.589	[−0.605, −0.573]
	300	Var_phase	−0.281	[−0.303, −0.258]	WE3_phase	−0.590	[−0.606, −0.574]	P2P_phase	−0.587	[−0.603, −0.571]
3	50	SC_rssi	−0.398	[−0.418, −0.378]	SE_phase	−0.055	[−0.079, −0.030]	Skew_rssi	−0.602	[−0.617, −0.586]
	100	SC_rssi	−0.379	[−0.399, −0.358]	Skew_rssi	−0.641	[−0.655, −0.627]	SE_phase	−0.061	[−0.085, −0.037]
	150	SC_rssi	−0.373	[−0.393, −0.351]	Skew_rssi	−0.657	[−0.670, −0.643]	SE_phase	−0.063	[−0.087, −0.038]
	200	SC_rssi	−0.369	[−0.390, −0.348]	Skew_rssi	−0.664	[−0.678, −0.651]	SE_phase	−0.065	[−0.089, −0.041]
	250	SC_rssi	−0.367	[−0.388, −0.346]	Skew_rssi	−0.669	[−0.682, −0.655]	SE_phase	−0.065	[−0.089, −0.041]
	300	SE_phase	−0.162	[−0.185, −0.138]	Skew_rssi	−0.672	[−0.685, −0.659]	Var_rssi	−0.041	[−0.066, −0.017]
4	50	Var_phase	0.766	[0.755, 0.775]	SC_rssi	0.033	[0.009, 0.058]	Kurt_rssi	−0.095	[−0.119, −0.070]
	100	SC_rssi	0.049	[0.024, 0.073]	P2P_rssi	0.220	[0.197, 0.244]	Slope_phase	0.441	[0.421, 0.460]
	150	SC_rssi	0.048	[0.024, 0.072]	P2P_rssi	0.227	[0.203, 0.250]	Slope_phase	0.450	[0.430, 0.469]
	200	SC_rssi	0.047	[0.023, 0.071]	P2P_rssi	0.229	[0.206, 0.252]	Slope_phase	0.454	[0.435, 0.474]
	250	SC_rssi	0.047	[0.023, 0.071]	P2P_rssi	0.231	[0.208, 0.254]	Slope_phase	0.456	[0.437, 0.476]
	300	Var_phase	0.799	[0.790, 0.807]	P2P_rssi	0.232	[0.209, 0.255]	SE_phase	0.221	[0.197, 0.244]

**Feature abbreviations:** Skew: Skewness; Var: Variance; P2P: Peak-to-Peak; ZCR: Zero-Crossing Rate; ACF1: Autocorrelation Lag-1; SC: Spectral Centroid; SE: Spectral Entropy; WE3: Wavelet Energy Level 3. Subscripts _rssi and _phase indicate the signal modality.

**Table 9:** Classification accuracy (%) for orientation estimation across different tags and sample sizes (*N*). Values represent the mean accuracy obtained from stratified 10-fold cross-validation.

Tag	Samples	Clustering			Classical ML					DL and Hybrid Architectures				
		K- Means	Agg	Mean Shift	Extra Trees	Random Forest	SVM	LGBM	XGBoost	ResNet	CNN	LSTM	CNN- LSTM	CNN+ SVM
1	50	99.00	98.78	100.00	100.00	100.00	100.00	100.00	100.00	100.00	99.94	100.00	100.00	100.00
	100	94.83	93.78	98.06	100.00	100.00	100.00	100.00	100.00	100.00	99.83	99.94	100.00	100.00
	150	96.50	98.00	97.89	100.00	100.00	100.00	100.00	100.00	100.00	99.94	99.50	100.00	99.94
	200	97.11	97.50	98.11	100.00	100.00	100.00	100.00	100.00	100.00	99.89	98.50	100.00	100.00
	250	96.83	96.50	98.33	100.00	100.00	100.00	100.00	100.00	100.00	100.00	99.61	100.00	99.94
	300	97.72	97.61	99.22	100.00	100.00	100.00	100.00	100.00	100.00	99.89	97.50	100.00	100.00
2	50	59.00	72.83	93.28	99.72	99.61	99.89	99.83	99.72	100.00	100.00	100.00	100.00	99.94
	100	97.39	97.50	97.33	99.78	99.61	99.89	99.78	99.67	100.00	99.94	100.00	100.00	100.00
	150	97.06	97.28	97.28	99.78	99.61	99.89	99.78	99.67	99.94	100.00	99.44	100.00	100.00
	200	96.72	97.00	97.17	99.78	99.61	99.89	99.78	99.67	100.00	100.00	91.72	99.94	100.00
	250	97.22	97.22	97.22	99.78	99.61	99.89	99.78	99.67	100.00	99.89	96.89	99.89	100.00
	300	97.44	97.44	97.44	99.78	99.61	99.89	99.83	99.67	100.00	99.39	90.83	100.00	100.00
3	50	88.50	88.72	99.44	100.00	100.00	100.00	100.00	100.00	100.00	99.33	100.00	100.00	100.00
	100	87.50	87.17	99.44	100.00	100.00	100.00	100.00	100.00	99.94	99.28	100.00	100.00	100.00
	150	88.94	86.89	99.78	100.00	100.00	100.00	100.00	100.00	100.00	98.72	99.94	100.00	99.72
	200	85.56	87.89	99.83	100.00	100.00	100.00	100.00	100.00	100.00	99.28	97.83	100.00	100.00
	250	86.06	87.33	99.72	100.00	100.00	100.00	100.00	100.00	100.00	98.78	98.83	100.00	100.00
	300	78.17	76.00	89.78	100.00	100.00	100.00	100.00	100.00	100.00	98.61	95.06	100.00	99.72
4	50	99.00	99.11	99.39	99.94	99.94	99.94	99.94	99.94	99.94	99.89	99.94	99.94	100.00
	100	74.28	77.50	78.17	99.94	99.94	99.94	99.94	99.94	100.00	99.33	99.94	99.94	100.00
	150	72.11	72.17	72.22	99.94	99.94	99.94	99.94	99.94	100.00	99.50	99.89	99.94	99.91
	200	72.06	72.17	72.22	99.94	99.94	99.94	99.94	99.94	99.83	98.61	99.89	100.00	99.78
	250	72.17	72.17	73.06	99.94	99.94	99.94	99.94	99.94	100.00	98.89	96.78	99.94	100.00
	300	79.89	80.06	70.89	99.94	99.94	99.94	99.94	99.94	100.00	97.39	95.17	100.00	100.00

**Table 10:** Experimental setup and objectives for model evaluation and deployment across the different stages of the study.

Exp.	Objective	Training Data	Test Data
EXP 1	Zero-Shot Evaluation	Lab (Controlled)	Shop floor (1 m)
EXP 2	Zero-Shot Evaluation	Lab (Controlled)	Shop floor (2 m)
EXP 3	Domain Adaptation	Shop floor (1 m)	Shop floor (1 m)
EXP 4	Cross-Distance	Shop floor (1 m)	Shop floor (2 m)
EXP 5	Unified Deployment	Shop floor (1 + 2 m)	Shop floor (1 + 2 m)

**Table 11:** Shop floor direction estimation accuracy (%) for the unified deployment scenario (EXP 5: Train and Test on combined 1 m and 2 m unstructured data).

Tag	Samples	EXP 5					
		Extra Trees	Random Forest	SVM	LGBM	XGBoost	CNN+ SVM
1	50	96.58	97.55	92.69	97.55	97.07	77.33
	100	97.55	97.08	91.68	97.07	97.07	74.87
	150	98.04	97.57	92.68	97.55	98.04	78.84
	200	97.54	97.57	92.20	96.56	97.55	77.78
	250	97.05	97.57	92.20	97.53	97.55	77.84
	300	97.54	97.57	92.68	97.53	97.06	81.29
2	50	86.27	83.35	76.43	85.20	88.62	59.61
	100	83.84	84.82	77.35	86.73	88.67	67.19
	150	84.86	84.36	77.40	87.28	89.14	67.19
	200	85.33	84.35	77.88	85.28	90.65	69.16
	250	85.84	82.38	76.90	88.17	88.21	73.94
	300	83.85	82.85	76.87	86.23	88.69	67.53
3	50	83.38	87.73	85.36	85.79	86.28	83.95
	100	85.80	89.26	84.85	85.76	87.76	86.83
	150	86.31	87.73	84.88	86.33	84.86	87.32
	200	86.29	88.27	85.38	84.29	87.33	86.82
	250	85.87	88.24	86.35	86.33	86.28	84.36
	300	86.36	87.75	84.91	85.80	86.30	84.88
4	50	98.05	98.05	98.05	97.56	98.04	93.12
	100	98.53	97.04	97.57	97.56	98.54	86.71
	150	98.05	97.06	98.05	98.04	98.54	91.11
	200	98.05	96.56	98.05	97.56	98.54	92.59
	250	98.05	96.56	98.05	98.04	98.54	91.14
	300	98.53	96.56	98.05	97.03	98.54	91.61

**Table 12:** Direction estimation accuracy breakdown by Gap Level (0 to 3) across all tags in the unified deployment (EXP 5). Values represent the Mean ± Standard Deviation across all evaluated sample sizes (N∈[50,300]). The *Sample Sensitivity* column indicates the window sizes that yielded the lowest (Worst) and highest (Best) resilience to signal fragmentation.

Tag	Model	Accuracy by Gap Level (%)				Sample Sensitivity (*N*)	
		Level 0 (Clean)	Level 1 (Minor)	Level 2 (Moderate)	Level 3 (Severe)	Worst	Best
1	Extra Trees	96.70 ± 0.60	98.25 ± 0.00	100.00 ± 0.00	98.69 ± 3.22	50	150
	Random Forest	97.36 ± 0.40	98.25 ± 0.00	100.00 ± 0.00	93.43 ± 3.22	50	≥150
	SVM	91.57 ± 0.59	92.98 ± 1.78	100.00 ± 0.00	92.11 ± 0.00	100	50
	LGBM	96.70 ± 0.32	97.65 ± 0.92	100.00 ± 0.00	100.00 ± 0.00	200	Var. *
	XGBoost	96.84 ± 0.49	97.65 ± 0.92	100.00 ± 0.00	100.00 ± 0.00	≤100	150
	CNN+SVM	78.35 ± 2.79	78.08 ± 2.27	70.98 ± 5.74	79.06 ± 4.50	50	300
2	Extra Trees	81.11 ± 1.57	89.74 ± 1.58	90.34 ± 3.00	93.44 ± 0.00	100	200
	Random Forest	79.04 ± 1.63	88.09 ± 1.45	94.21 ± 0.00	93.44 ± 0.00	250	150
	SVM	72.96 ± 0.69	80.75 ± 0.06	82.25 ± 3.75	92.01 ± 2.84	50	200
	LGBM	83.08 ± 1.65	89.45 ± 2.08	97.09 ± 3.19	91.21 ± 3.45	50	100
	XGBoost	86.15 ± 1.25	91.98 ± 0.81	100.00 ± 0.00	88.88 ± 3.53	50	200
	CNN+SVM	67.13 ± 6.54	75.37 ± 1.88	62.71 ± 11.62	47.84 ± 9.88	50	250
3	Extra Trees	92.87 ± 0.92	86.54 ± 2.07	79.46 ± 6.42	72.39 ± 1.85	50	300
	Random Forest	94.02 ± 0.55	91.80 ± 0.00	82.67 ± 3.35	74.05 ± 2.14	150	100
	SVM	92.33 ± 0.84	84.24 ± 1.48	84.09 ± 0.00	71.58 ± 1.45	150	250
	LGBM	93.88 ± 1.18	89.46 ± 1.51	80.62 ± 2.93	66.08 ± 3.06	200	250
	XGBoost	94.04 ± 1.33	91.23 ± 1.03	80.73 ± 2.91	67.31 ± 1.94	150	200
	CNN+SVM	91.07 ± 1.39	92.61 ± 2.05	83.69 ± 5.13	66.47 ± 4.68	50	200
4	Extra Trees	98.53 ± 0.46	100.00 ± 0.00	95.99 ± 0.00	92.35 ± 0.00	Var. *	100, 300
	Random Forest	96.88 ± 0.75	100.00 ± 0.00	97.32 ± 2.07	84.64 ± 0.00	≥200	50
	SVM	98.09 ± 0.35	100.00 ± 0.00	95.99 ± 0.00	92.35 ± 0.00	100	50, ≥150
	LGBM	98.08 ± 0.37	100.00 ± 0.00	93.34 ± 2.05	92.29 ± 0.00	300	150, 250
	XGBoost	98.23 ± 0.00	100.00 ± 0.00	95.99 ± 0.00	98.72 ± 3.15	50	≥100
	CNN+SVM	92.41 ± 3.60	90.61 ± 2.31	87.91 ± 4.43	87.19 ± 4.00	200	50

* Var.: Performance peaked/bottomed uniformly across multiple sample sizes (e.g., N={50,150,250}).

## Data Availability

The original contributions presented in this study are included in the article. The code used for preprocessing, augmentation, and model training is available from the corresponding author upon reasonable request.

## Appendix A

This appendix provides supplementary visualizations of the raw RSSI and Phase profiles acquired during the experimental setup, prior to any temporal resampling or filtering. These figures empirically corroborated the feature selection and statistical analyses discussed in Section 4.1.1 and Section 4.2.1.

Figure A1 illustrates the temporal evolution of the signals during distinct directional movements (Left vs. Right). The prominent asymmetric patterns, particularly the rising/falling slopes of Phase for Tags 2 and 3, confirmed the strong directional dependencies identified by the mRMR algorithm.

Furthermore, Figure A2 presents the value distributions as a function of box orientation. While Phase values remain broadly distributed across the [−π,π] interval, RSSI distributions exhibit noticeable shifts between orientations (ranging from approximately −47 dBm to −77 dBm). The presence of outliers, notably for Tags 2 and 3, highlighted the multipath interference and NLoS conditions that motivate the robust preprocessing and domain randomization strategies adopted in this work.

## Appendix B

This appendix reports the complete macro-averaged classification metrics for both direction and orientation estimation tasks. Metrics are computed as the mean across all four tags for each sample size *N*, obtained from stratified 10-fold cross-validation. These results complement the accuracy analysis discussed in Section 4.1 and Section 4.2, confirming that Accuracy, Precision, Recall, and F1-Score followed consistent trends across all configurations. Table A1 summarizes the results for the direction estimation task. Table A2 reports the equivalent metrics for the orientation estimation task (three-class problem). The macro-average weighting ensures equal contribution from all three orientations, consistent with the balanced class distribution of the dataset.

**Table A1:** Macro-averaged Accuracy (Acc), Precision (Pre), Recall (Rec), and F1-Score for direction estimation. Values represent the mean across all four tags and are obtained from stratified 10-fold cross-validation.

Tag	Samples	Clustering			Classical ML					DL and Hybrid Architectures				
		K- Means	Agg	Mean Shift	Extra Trees	Random Forest	SVM	LGBM	XGBoost	ResNet	CNN	LSTM	CNN- LSTM	CNN+ SVM
Acc	50	95.96	94.88	89.54	99.92	99.96	99.92	100.00	99.92	85.29	99.21	99.92	100.00	100.00
	100	96.00	95.50	90.58	99.96	99.96	99.92	99.88	99.92	86.42	98.67	99.96	99.17	100.00
	150	96.29	93.75	92.25	99.96	99.96	99.92	99.88	99.92	91.13	98.96	98.79	100.00	99.95
	200	98.83	97.50	91.83	99.96	99.96	99.92	99.88	99.92	89.92	95.92	99.17	97.83	99.93
	250	98.92	98.21	90.00	99.96	99.96	99.92	99.88	99.92	87.13	97.08	98.29	98.50	99.93
	300	98.58	97.00	91.33	99.96	99.96	99.92	99.88	99.92	93.33	95.21	96.00	96.21	99.93
Pre	50	96.00	95.02	87.12	99.92	99.96	99.92	100.00	99.92	80.08	99.49	99.92	100.00	100.00
	100	95.44	94.99	87.91	99.96	99.96	99.92	99.88	99.92	80.67	99.07	99.96	99.50	100.00
	150	95.87	94.55	92.24	99.96	99.96	99.92	99.88	99.92	87.84	99.38	99.24	100.00	99.96
	200	98.86	97.76	89.74	99.96	99.96	99.92	99.88	99.92	85.56	97.57	99.34	97.60	99.93
	250	98.95	98.29	85.66	99.96	99.96	99.92	99.88	99.92	82.63	96.93	98.33	98.70	99.93
	300	98.64	97.07	88.55	99.96	99.96	99.92	99.88	99.92	91.36	94.63	95.83	94.33	99.93
Rec	50	95.92	94.88	89.54	99.92	99.96	99.92	100.00	99.92	85.29	99.21	99.92	100.00	100.00
	100	96.00	95.50	90.58	99.96	99.96	99.92	99.88	99.92	86.42	98.67	99.96	99.17	100.00
	150	95.58	93.75	92.25	99.96	99.96	99.92	99.88	99.92	91.13	98.96	98.79	100.00	99.95
	200	98.83	97.50	91.83	99.96	99.96	99.92	99.88	99.92	89.92	95.92	99.17	97.83	99.93
	250	98.92	98.21	90.00	99.96	99.96	99.92	99.88	99.92	87.13	97.08	98.29	98.50	99.93
	300	98.58	97.00	91.33	99.96	99.96	99.92	99.88	99.92	93.33	95.21	96.00	96.21	99.93
F1	50	95.84	94.74	87.25	99.92	99.96	99.92	100.00	99.92	80.58	99.13	99.92	100.00	100.00
	100	95.56	95.05	88.39	99.96	99.96	99.92	99.87	99.92	82.00	98.59	99.96	99.06	100.00
	150	95.48	93.52	91.16	99.96	99.96	99.92	99.87	99.92	88.52	98.79	98.60	100.00	99.95
	200	98.83	97.46	90.09	99.96	99.96	99.92	99.87	99.92	86.92	95.12	99.14	97.27	99.93
	250	98.92	98.20	87.08	99.96	99.96	99.92	99.87	99.92	83.52	96.50	98.29	98.38	99.93
	300	98.58	96.99	89.20	99.96	99.96	99.92	99.87	99.92	91.54	94.14	95.54	94.96	99.93

**Table A2:** Macro-averaged Accuracy (Acc), Precision (Pre), Recall (Rec), and F1-Score for orientation estimation. Values represent the mean across all four tags and are obtained from stratified 10-fold cross-validation.

Tag	Samples	Clustering			Classical ML					DL and Hybrid Architectures				
		K- Means	Agg	Mean Shift	Extra Trees	Random Forest	SVM	LGBM	XGBoost	ResNet	CNN	LSTM	CNN- LSTM	CNN+ SVM
Acc	50	86.38	89.86	98.03	99.92	99.89	99.96	99.94	99.92	99.99	99.79	99.99	99.99	99.99
	100	88.50	88.99	93.25	99.93	99.89	99.96	99.93	99.90	99.99	99.60	99.97	99.99	100.00
	150	88.65	88.58	91.79	99.93	99.89	99.96	99.93	99.90	99.99	99.54	99.69	99.99	99.89
	200	87.86	88.64	91.83	99.93	99.89	99.96	99.93	99.90	99.96	99.44	96.99	99.99	99.94
	250	88.07	88.31	92.08	99.93	99.89	99.96	99.93	99.90	100.00	99.39	98.03	99.96	99.99
	300	88.31	87.78	89.33	99.93	99.89	99.96	99.94	99.90	100.00	98.82	94.64	100.00	99.93
Pre	50	87.85	91.48	98.24	99.92	99.89	99.96	99.95	99.92	99.99	99.80	99.99	99.99	99.99
	100	90.30	91.45	94.41	99.93	99.89	99.96	99.93	99.91	99.99	99.62	99.97	99.99	100.00
	150	90.91	91.24	93.20	99.93	99.89	99.96	99.93	99.91	99.99	99.56	99.71	99.99	99.89
	200	90.50	91.17	93.21	99.93	99.89	99.96	99.93	99.91	99.96	99.50	97.11	99.99	99.94
	250	90.66	90.93	93.47	99.93	99.89	99.96	99.93	99.91	100.00	99.42	98.10	99.96	99.99
	300	90.74	89.99	90.22	99.93	99.89	99.96	99.95	99.91	100.00	99.00	95.36	100.00	99.93
Rec	50	86.38	89.86	98.03	99.92	99.89	99.96	99.94	99.92	99.99	99.79	99.99	99.99	99.99
	100	88.50	88.99	93.25	99.93	99.89	99.96	99.93	99.90	99.99	99.60	99.97	99.99	100.00
	150	88.65	88.58	91.79	99.93	99.89	99.96	99.93	99.90	99.99	99.54	99.69	99.99	99.89
	200	87.86	88.64	91.83	99.93	99.89	99.96	99.93	99.90	99.96	99.44	96.99	99.99	99.94
	250	88.07	88.31	92.08	99.93	99.89	99.96	99.93	99.90	100.00	99.39	98.03	99.96	99.99
	300	88.31	87.78	89.33	99.93	99.89	99.96	99.94	99.90	100.00	98.82	94.64	100.00	99.93
F1	50	86.01	89.50	98.04	99.92	99.89	99.96	99.94	99.92	99.99	99.79	99.99	99.99	99.99
	100	87.81	88.55	93.06	99.93	99.89	99.96	99.93	99.90	99.99	99.60	99.97	99.99	100.00
	150	88.23	87.97	91.55	99.93	99.89	99.96	99.93	99.90	99.99	99.54	99.69	99.99	99.89
	200	87.30	88.10	91.60	99.93	99.89	99.96	99.93	99.90	99.96	99.44	96.97	99.99	99.94
	250	87.56	87.76	91.85	99.93	99.89	99.96	99.93	99.90	100.00	99.39	98.01	99.96	99.99
	300	86.91	86.30	87.28	99.93	99.89	99.96	99.94	99.90	100.00	98.79	94.54	100.00	99.93

## Appendix C

This appendix summarizes the computational latency and static model size for all models in both laboratory and shop floor settings. Table A3 presents the mean inference time and worst-case (peak) latency for all tested window sizes (N∈[50,300]).

**Table A3:** Inference latency (μs) for all models, reported as Mean (Absolute Peak) of all sample sizes and the maximum model size observed across all tags.

Model	Lab: Direction		Lab: Orientation		Shop Floor (EXP 5)	
	Latency (μs)	Model Size (KB)	Latency (μs)	Model Size (KB)	Latency (μs)	Model Size (KB)
K-Means	7.45 (8.23)	0.88	1.39 (1.56)	1.39	—	—
Agglomerative	0.01 (0.04)	1.94	0.01 (0.07)	4.75	—	—
Mean Shift	2.90 (3.58)	1.01	1.48 (1.90)	2.02	—	—
Extra Trees	712.44 (883.51)	3004	156.03 (179.29)	7582	130.77 (135.59)	7184
Random Forest	1117.45 (1417.57)	2782	382.11 (457.31)	7125	213.28 (220.71)	8356
SVM	83.87 (140.10)	2478	290.75 (601.44)	9032	175.00 (358.66)	6931
LGBM	18.53 (27.50)	233	46.40 (58.64)	2025	8.48 (9.97)	286
XGBoost	115.59 (140.77)	520	92.60 (123.44)	2136	130.06 (149.40)	885
ResNet	3547.19 (4659.04)	710	1297.53 (1417.94)	711	—	—
CNN	1881.31 (2457.86)	109	718.50 (1666.41)	107	—	—
LSTM	6848.91 (11,597.39)	826	2074.94 (2478.69)	828	—	—
CNN-LSTM	6059.90 (9202.25)	402	2466.18 (4193.59)	1323	—	—
CNN+SVM	17.59 (64.06)	150	24.41 (66.73)	147	366.43 (804.85)	970

## Appendix D

This appendix provides the comprehensive empirical data supporting the progressive shop floor evaluation framework discussed in Section 4.3. Table A4 quantifies the performance observed when models optimized with laboratory data are implemented in industrial environments without adaptation (EXP 1 and EXP 2). Table A5 presents the results of the 1 m domain adaptation (EXP 3) and the model’s inability to generalize to a 2 m spatial constraint without dedicated spatial training (EXP 4).

**Table A4:** Average accuracy (%) achieved in estimating direction on the shop floor for the Zero-Shot deployment scenario (EXP 1 and EXP 2).

Tag	Samples	EXP 1						EXP 2					
		Extra Trees	Random Forest	SVM	LGBM	XGBoost	CNN+ SVM	Extra Trees	Random Forest	SVM	LGBM	XGBoost	CNN+ SVM
1	50	75.84	81.03	68.03	77.65	72.50	70.75	86.47	76.24	88.71	71.71	68.34	44.01
	100	74.96	75.08	69.79	81.07	69.86	70.64	85.37	71.76	87.60	61.43	64.86	43.00
	150	69.76	75.90	69.82	80.24	69.84	78.38	84.25	66.01	87.57	62.58	66.01	46.35
	200	69.78	75.00	68.94	77.54	68.17	74.97	84.25	66.01	88.71	62.58	66.01	49.83
	250	76.74	75.90	69.79	74.02	70.69	87.03	84.21	66.01	87.57	64.94	67.17	46.31
	300	71.50	73.37	69.79	76.65	68.98	88.80	85.37	64.86	87.57	59.20	67.17	46.24
2	50	71.63	72.38	71.51	69.79	74.00	53.34	64.83	63.68	60.28	62.50	66.02	49.93
	100	72.47	70.69	70.59	74.10	75.77	60.35	62.54	64.73	56.84	65.90	66.02	52.34
	150	70.69	71.57	68.07	73.23	72.35	75.00	63.59	63.71	54.46	64.91	64.87	50.00
	200	74.18	71.45	68.90	72.46	70.62	63.02	63.69	63.85	55.58	65.90	67.09	50.00
	250	70.72	74.07	67.12	71.57	76.70	49.91	63.63	61.69	55.58	69.38	65.94	54.57
	300	71.56	70.71	67.14	70.58	74.05	77.50	62.46	60.44	55.58	67.19	65.94	50.00
3	50	54.94	54.15	73.16	49.09	51.63	47.47	49.03	45.63	49.11	44.40	44.44	39.78
	100	54.13	55.02	73.90	53.34	49.94	39.71	49.13	44.45	49.19	44.51	46.83	46.62
	150	54.13	55.04	72.20	51.61	50.79	44.64	47.84	43.31	48.02	45.69	45.78	40.99
	200	54.13	54.99	73.92	52.48	51.63	21.46	47.87	44.45	46.93	45.72	44.56	36.38
	250	53.24	54.13	73.05	51.61	50.74	19.83	47.83	44.45	48.06	42.28	48.01	46.64
	300	54.13	52.48	72.17	53.37	51.57	31.86	47.89	44.45	46.87	46.81	47.94	46.57
4	50	50.00	50.00	50.00	49.12	48.30	50.00	85.08	78.36	82.90	76.09	71.59	61.24
	100	50.00	50.00	50.00	49.12	49.98	50.85	78.36	77.26	81.71	73.87	70.46	65.86
	150	50.00	50.00	50.00	50.00	49.12	52.56	79.46	77.26	82.84	75.02	72.77	56.71
	200	50.00	51.72	50.00	48.30	49.12	50.85	79.49	73.93	83.97	73.87	70.46	70.24
	250	50.00	51.73	50.00	49.12	49.12	50.00	78.36	72.84	81.71	73.87	72.77	68.13
	300	50.00	54.34	50.00	49.12	48.30	50.00	78.36	76.14	79.43	75.02	69.34	57.94

**Table A5:** Average accuracy (%) of direction estimation illustrating spatial vulnerability, contrasting the successful Domain Adaptation scenario (EXP 3) with Cross-Distance evaluation (EXP 4).

Tag	Samples	EXP 3						EXP 4					
		Extra Trees	Random Forest	SVM	LGBM	XGBoost	CNN+ SVM	Extra Trees	Random Forest	SVM	LGBM	XGBoost	CNN+ SVM
1	50	95.69	98.28	96.56	98.26	97.36	94.89	50.00	50.00	50.00	50.00	50.00	41.02
	100	99.15	98.28	96.56	98.26	97.39	92.29	50.00	50.00	50.00	50.00	50.00	43.18
	150	98.31	98.28	95.69	97.39	97.39	94.81	50.00	50.00	50.00	50.00	50.00	51.11
	200	97.37	98.28	95.71	97.39	98.28	94.88	50.00	50.00	50.00	50.00	50.00	31.86
	250	98.26	98.28	96.56	97.39	98.25	94.84	50.00	50.00	50.00	50.00	50.00	44.26
	300	96.56	98.28	95.71	96.50	97.39	96.56	50.00	50.00	50.00	50.00	50.00	49.86
2	50	87.97	85.38	82.69	86.99	88.67	63.92	71.56	72.66	62.43	70.60	69.36	33.01
	100	88.81	83.60	80.11	86.98	88.77	80.20	75.12	71.56	63.56	75.29	71.49	39.89
	150	87.89	82.76	80.91	87.05	88.76	81.15	78.36	71.57	64.69	77.35	76.16	50.00
	200	89.65	83.60	80.13	88.84	87.08	75.13	82.86	73.84	62.32	67.17	68.24	50.00
	250	89.64	83.61	80.06	87.08	90.42	79.46	76.34	72.76	64.56	63.71	71.51	50.00
	300	87.97	83.60	80.98	89.69	87.81	70.90	79.56	77.23	64.56	70.44	72.71	50.00
3	50	96.51	97.38	94.79	96.57	97.38	96.54	50.00	50.00	50.00	50.00	50.00	52.26
	100	97.38	97.38	94.79	96.57	97.38	97.38	50.00	50.00	50.00	50.00	50.00	49.94
	150	97.38	96.57	94.79	96.57	97.38	97.38	50.00	50.00	50.00	50.00	50.00	49.89
	200	96.51	96.57	94.79	96.57	97.38	95.72	50.00	50.00	50.00	50.00	50.00	48.92
	250	97.38	96.57	94.79	96.57	97.38	96.54	50.00	50.00	50.00	50.00	50.00	46.52
	300	97.38	96.57	94.79	97.38	97.38	94.00	50.00	50.00	50.00	50.00	50.00	41.97
4	50	98.28	96.58	99.16	97.43	98.28	85.36	72.64	86.18	56.74	84.01	83.91	50.00
	100	99.16	98.28	97.47	98.28	98.28	89.64	71.50	86.18	53.39	82.96	81.70	53.43
	150	99.16	97.44	98.30	98.28	98.28	89.64	77.30	86.29	56.80	82.92	85.02	57.90
	200	99.16	98.28	98.30	98.28	98.28	92.27	74.89	85.16	55.61	82.92	85.02	65.91
	250	99.16	97.44	99.16	98.28	98.28	93.12	77.12	86.29	55.61	88.50	87.36	62.37
	300	99.16	98.28	98.30	98.28	98.28	84.49	79.46	85.08	54.54	79.48	85.02	56.75

To ensure that the unified deployment architecture (EXP 5) did not favor one of the spatial constraints, Table A6 shows the results of the models trained on the combined unstructured dataset (1 m + 2 m) and tested separately on 1 m and 2 m.

**Table A6:** Average accuracy (%) achieved in direction estimation where the 1 m and 2 m test subsets were evaluated independently (EXP 5—Unified Deployment Ablation).

Tag	Samples	1 m						2 m					
		Extra Trees	Random Forest	SVM	LGBM	XGBoost	CNN+ SVM	Extra Trees	Random Forest	SVM	LGBM	XGBoost	CNN+ SVM
1	50	95.74	97.45	91.46	97.45	96.60	91.37	97.69	97.69	94.31	97.69	97.69	58.84
	100	97.45	96.62	90.59	96.60	96.60	88.82	97.69	97.69	93.12	97.69	97.69	56.48
	150	98.31	96.62	92.31	97.45	97.45	90.58	97.69	98.81	93.16	97.69	98.81	63.38
	200	97.43	96.62	91.48	95.71	97.45	89.64	97.69	98.81	93.16	97.69	97.69	62.15
	250	96.56	96.62	91.48	96.56	97.45	89.68	97.69	98.81	93.16	98.81	97.69	62.24
	300	97.43	96.62	92.31	96.56	96.59	91.39	97.69	98.81	93.16	98.81	97.69	67.98
2	50	88.73	82.74	73.19	83.50	88.70	54.23	88.73	82.74	73.19	83.50	88.70	66.71
	100	84.43	86.15	77.47	85.24	86.14	65.66	84.43	86.15	77.47	85.24	86.14	69.20
	150	86.16	84.51	75.76	85.32	86.12	74.26	86.16	84.51	75.76	85.32	86.12	57.88
	200	87.86	86.20	75.75	80.93	87.87	75.09	87.86	86.20	75.75	80.93	87.87	61.35
	250	87.02	84.43	74.94	86.07	87.06	79.28	87.02	84.43	74.94	86.07	87.06	66.90
	300	84.42	86.16	74.02	82.69	86.17	78.39	84.42	86.16	74.02	82.69	86.17	53.21
3	50	96.51	97.38	97.38	96.57	95.64	94.04	66.07	75.00	69.53	71.59	73.95	70.65
	100	97.38	98.25	96.52	96.50	96.48	95.70	70.54	77.40	69.48	71.60	76.25	75.14
	150	97.38	98.25	97.38	96.56	94.82	96.48	71.71	73.85	68.40	72.85	71.74	75.23
	200	97.38	97.43	96.52	96.50	95.68	96.55	71.67	76.19	70.69	68.19	76.32	74.00
	250	97.38	98.25	97.38	94.86	94.77	95.67	70.68	75.04	71.82	75.09	75.09	69.44
	300	97.38	97.44	96.52	95.65	95.66	93.14	71.84	74.99	69.62	72.81	73.97	73.99
4	50	98.30	97.43	98.30	96.58	97.43	94.83	97.71	98.86	97.71	98.85	98.85	90.86
	100	99.16	96.53	97.47	96.58	98.30	85.34	97.71	97.71	97.71	98.85	98.85	88.52
	150	98.30	95.69	98.30	97.43	98.30	93.04	97.71	98.86	97.71	98.85	98.85	88.57
	200	98.30	95.69	98.30	96.58	98.30	94.82	97.71	97.71	97.71	98.85	98.85	89.65
	250	98.30	95.69	98.30	97.43	98.30	94.86	97.71	97.71	97.71	98.85	98.85	86.23
	300	99.16	95.69	98.30	95.65	98.30	94.79	97.71	97.71	97.71	98.85	98.85	87.41

## Appendix E

Table A7 reports the per-direction classification accuracy achieved under the unified deployment configuration (EXP 5), in which models were trained on the combined 1 m and 2 m shop floor dataset and evaluated independently on each distance subset.

**Table A7:** Per-direction classification accuracy (%) under unified domain adaptation (EXP 5—Unified Deployment Ablation), with the 1 m and 2 m test subsets evaluated independently. Right and left correspond to the two opposing movement directions along the conveyor axis.

Tag	Samples	Right						Left					
		Extra Trees	Random Forest	SVM	LGBM	XGBoost	CNN+ SVM	Extra Trees	Random Forest	SVM	LGBM	XGBoost	CNN+ SVM
1	50	97.07	97.09	91.28	97.09	96.13	75.44	96.09	98.00	94.09	98.00	98.00	79.22
	100	97.09	97.09	90.22	96.13	96.13	75.48	98.00	97.07	93.14	98.00	98.00	74.25
	150	98.08	97.09	91.26	97.09	97.09	76.40	98.00	98.04	94.09	98.00	98.97	81.28
	200	97.07	97.09	90.31	95.12	97.09	81.25	98.00	98.04	94.09	98.00	98.00	74.30
	250	96.08	97.09	90.31	96.08	97.09	75.45	98.00	98.04	94.09	98.97	98.00	80.23
	300	97.07	97.09	91.26	96.08	96.12	81.41	98.00	98.04	94.09	98.97	98.00	81.17
2	50	84.26	80.28	76.36	83.20	88.07	81.03	88.27	86.41	76.50	87.18	89.16	38.18
	100	80.28	81.29	76.29	84.27	87.11	86.21	87.39	88.33	78.40	89.18	90.22	48.16
	150	80.38	81.29	74.40	88.22	88.05	85.23	89.33	87.41	80.39	86.34	90.23	49.15
	200	80.30	79.36	75.40	85.26	90.13	90.12	90.35	89.33	80.36	85.29	91.15	48.18
	250	81.36	76.42	74.36	89.14	88.08	79.27	90.30	88.33	79.44	87.18	88.33	68.60
	300	78.38	77.41	74.36	87.13	87.09	74.40	89.30	88.28	79.38	85.32	90.27	60.65
3	50	74.43	82.21	78.41	79.32	84.15	82.29	92.31	93.24	92.31	92.25	88.41	85.61
	100	77.33	84.24	78.36	81.20	84.15	83.28	94.27	94.27	91.33	90.32	91.35	90.36
	150	79.37	83.20	78.36	81.34	81.29	84.18	93.24	92.25	91.39	91.32	88.43	90.44
	200	77.38	83.20	79.41	76.26	84.23	85.15	95.19	93.33	91.33	92.31	90.41	88.48
	250	78.45	83.20	80.38	82.26	82.16	85.12	93.27	93.27	92.31	90.40	90.40	83.58
	300	78.45	82.27	79.41	82.27	82.18	80.33	94.27	93.23	90.40	89.32	90.41	89.42
4	50	97.05	98.04	97.05	97.07	98.03	89.18	99.03	98.04	99.03	98.04	98.04	97.05
	100	98.02	96.03	97.05	97.07	98.03	74.38	99.03	98.04	98.09	98.04	99.03	99.03
	150	97.05	97.02	97.05	98.03	98.03	88.11	99.03	97.08	99.03	98.04	99.03	94.11
	200	97.05	96.03	97.05	97.07	98.03	86.13	99.03	97.08	99.03	98.04	99.03	99.03
	250	97.05	96.03	97.05	98.03	98.03	83.23	99.03	97.08	99.03	98.04	99.03	99.03
	300	98.02	96.03	97.05	96.02	98.03	86.14	99.03	97.08	99.03	98.04	99.03	97.06
